# Extracellular DNA, cell surface proteins and c-di-GMP promote biofilm formation in *Clostridioides difficile*

**DOI:** 10.1038/s41598-020-78437-5

**Published:** 2021-02-05

**Authors:** Lisa F. Dawson, Johann Peltier, Catherine L. Hall, Mark A. Harrison, Maria Derakhshan, Helen A. Shaw, Neil F. Fairweather, Brendan W. Wren

**Affiliations:** 1 Department of Infection Biology, London School of Hygiene and Tropical Medicine, London, UK; 2 Department of Life Sciences, Imperial College London, South Kensington Campus, London, SW7 2AZ UK; 3 Present Address: Université Paris-Saclay, CEA, CNRS, Institute for Integrative Biology of the Cell (I2BC), 91198 Gif-sur-Yvette, France; 4 Present Address: National Institute for Biological Standards and Control, Potters Bar, UK

**Keywords:** Antimicrobials, Bacteria, Bacteriology, Biofilms, Microbial communities, Microbial genetics, Microbiology, Molecular biology

## Abstract

*Clostridioides difficile* is the leading cause of nosocomial antibiotic-associated diarrhoea worldwide, yet there is little insight into intestinal tract colonisation and relapse. In many bacterial species, the secondary messenger cyclic-di-GMP mediates switching between planktonic phase, sessile growth and biofilm formation. We demonstrate that c-di-GMP promotes early biofilm formation in *C. difficile* and that four cell surface proteins contribute to biofilm formation, including two c-di-GMP regulated; CD2831 and CD3246, and two c-di-GMP-independent; CD3392 and CD0183. We demonstrate that *C. difficile* biofilms are composed of extracellular DNA (eDNA), cell surface and intracellular proteins, which form a protective matrix around *C. difficile* vegetative cells and spores, as shown by a protective effect against the antibiotic vancomycin. We demonstrate a positive correlation between biofilm biomass, sporulation frequency and eDNA abundance in all five *C. difficile* lineages. Strains 630 (RT012), CD305 (RT023) and M120 (RT078) contain significantly more eDNA in their biofilm matrix than strains R20291 (RT027) and M68 (RT017). DNase has a profound effect on biofilm integrity, resulting in complete disassembly of the biofilm matrix, inhibition of biofilm formation and reduced spore germination. The addition of exogenous DNase could be exploited in treatment of *C. difficile* infection and relapse, to improve antibiotic efficacy.

## Introduction

*Clostridiodes difficile* (formerly *Clostridium difficile*) is a spore-forming obligate anaerobe, responsible for the majority of nosocomial antibiotic-associated diarrhoea cases worldwide. Treatment with antibiotics for an underlying condition alters the intestinal microbiota, which results in susceptibility to colonisation with *C. difficile*^1^. Ingested spores, the etiological agent of *C. difficile* transmission, germinate in the small intestine upon exposure to conjugated and deconjugated bile salts^2^, resulting in outgrowth of metabolically active vegetative cells, which produce toxins, TcdA and TcdB, responsible for damage of the host intestinal tract^3^. Current data indicates that 15–35% of patients suffering from *C. difficile* infection (CDI) relapse within two months post-treatment^4^, with increasing probability of relapse thereafter, indicating the presence of a potential reservoir of *C. difficile* re-seeding the intestinal tract of susceptible patients^5^.

Many pathogenic bacteria aggregate and form biofilms, defined as communities of bacteria encased within a self-produced matrix, which provide a protective barrier against host defenses and antimicrobials^6^. Biofilm formation allows pathogens to attain a foothold in a given environment, such as a tissue or on an implanted device^7^, which facilitate recurrent bacterial infections for various pathogens^8–10^. Biofilm formation is a multifaceted process, divided into three stages; (1) attachment, (2) maturation and (3) dispersal^11^. Active dispersal, in which planktonic cells are released from a biofilm matrix facilitates colonization of a subsequent niche^12^ and contributes to disease recurrence^8–10^. The structure and composition of a biofilm can vary between species, however, generally the biofilm matrix is comprised of a self-produced Extracellular Polymeric Substance (EPS), usually formed in three primary categories: (1) exopolysaccharide, (2) extracellular and cell surface proteins and (3) extracellular DNA (eDNA)^13^. Production and composition of the biofilm matrix within a species can depend on environmental triggers^11,14^, for example, the opportunistic pathogen *Pseudomonas aeruginosa* produces either a mucoid biofilm, with alginate as a key EPS component, or a non-mucoid biofilm, with the Psl and Pel exopolysaccharides as the predominant matrix proteins^11,15,16^. Despite the presence of species-specific exopolysaccharides, eDNA forms a crucial part of the biofilm structure in a number of species, including *P. aeruginosa*^17,18^*, Staphylococcus aureus*^19,20^*, Listeria monocytogenes*^21^, and *Streptococcus pneumoniae*^22^.

To date, several studies have shown that *C. difficile* forms biofilms; including the formation of multicellular structures in vitro^23–29^, dual species biofilms^30^, multispecies biofilms in a human chemostat^31,32^, and the formation of mats and aggregates in vivo^33–35^. There is evidence that *C. difficile* biofilms are composed of DNA and protein surrounding adherent spores and vegetative cells, although the extent to which they contribute to biofilm formation, biofilm integrity and dispersal remains to be determined^23–25,29^. *C. difficile* genome searches have failed to identify putative genes encoding biofilm matrix proteins. In fact, most factors and components required by other biofilm-producing bacteria are not conserved in *C. difficile*^28^. Several genes have been attributed to biofilm formation in *C. difficile*, most notably, the sporulation master regulator *spo0A*^23,25,29^, the quorum sensing regulator *luxS*^36,37^, and the germination receptor *sleC*^25^, whose mutations significantly decrease biofilm formation. Conversely, inactivation of the chaperones *dnaK* and *hfq*^38,39^, the transcriptional regulators *CD2214–CD2215* (double mutant)^28^ and the regulator of the SOS response network *lexA*^40^ increased biofilm formation. Mutation of genes encoding the cell surface protease *cwp84* and the flagella filament *fliC* have been shown to both positively^36,41^ and negatively^26,36^ impact biofilm biomass. This highlights that biofilm formation in *C. difficile* like other species is a multifaceted complex process.

In *C. difficile* and other bacteria, the secondary messenger c-di-GMP enhances biofilm formation and aggregation by inversely regulating aggregation and cell motility via the modulation of flagella and type IV pili expression^42–44^. In other species c-di-GMP also regulates production of extracellular polysaccharides^45,46^ and surface associated cell wall proteins^47^, which form part of the biofilm matrix, as well as modulating the switch between motile and sessile lifestyle. Inactivation of the phosphodiesterase PcdA, which facilitates turnover of c-di-GMP, increases biofilm formation in *C. difficile*^44^. *C. difficile* encodes a number of cell surface associated proteins, including those covalently anchored to peptidoglycan within the cell wall via the single sortase, SrtB^48–51^. CD2831 and CD3246 are cell surface proteins, which are cleaved and covalently anchored to *m*-DAP in the peptidoglycan by SrtB^50,52–54^. Under low-level c-di-GMP, the protease PPEP-1 is produced which cleaves CD2831 and CD3246 from the peptidoglycan^55,56^. Conversely under elevated levels of c-di-GMP, PPEP-1 is repressed and *CD2831* is highly expressed, leading to the surface exposure of covalently anchored CD2831^49^ and CD3246^54,55^.

We investigated factors involved in biofilm formation in *C. difficile* including biomass structure, production and stability. We elucidated the role of c-di-GMP and four cell surface associated proteins, CD0183, CD2831, CD3246 and CD3392 in early biofilm formation in *C. difficile* and highlight the importance of eDNA in biofilm development, integrity and resistance to the anti-*C. difficile* antibiotic vancomycin. We identified that all five *C. difficile* lineages are dependent on eDNA for biofilm initiation, stability and biomass, which positively correlates with sporulation frequency. Herein, we present a model of biofilm formation, and a strategy to interfere with (1) attachment and (2) biofilm maturation for *C. difficile*, which potentially provides a non-microbicidal strategy to combine with current antibiotic therapies to reduce the reservoir of *C. difficile*.

## Results

### Visualization and composition of *C. difficile* biofilms

**Figure 1:**
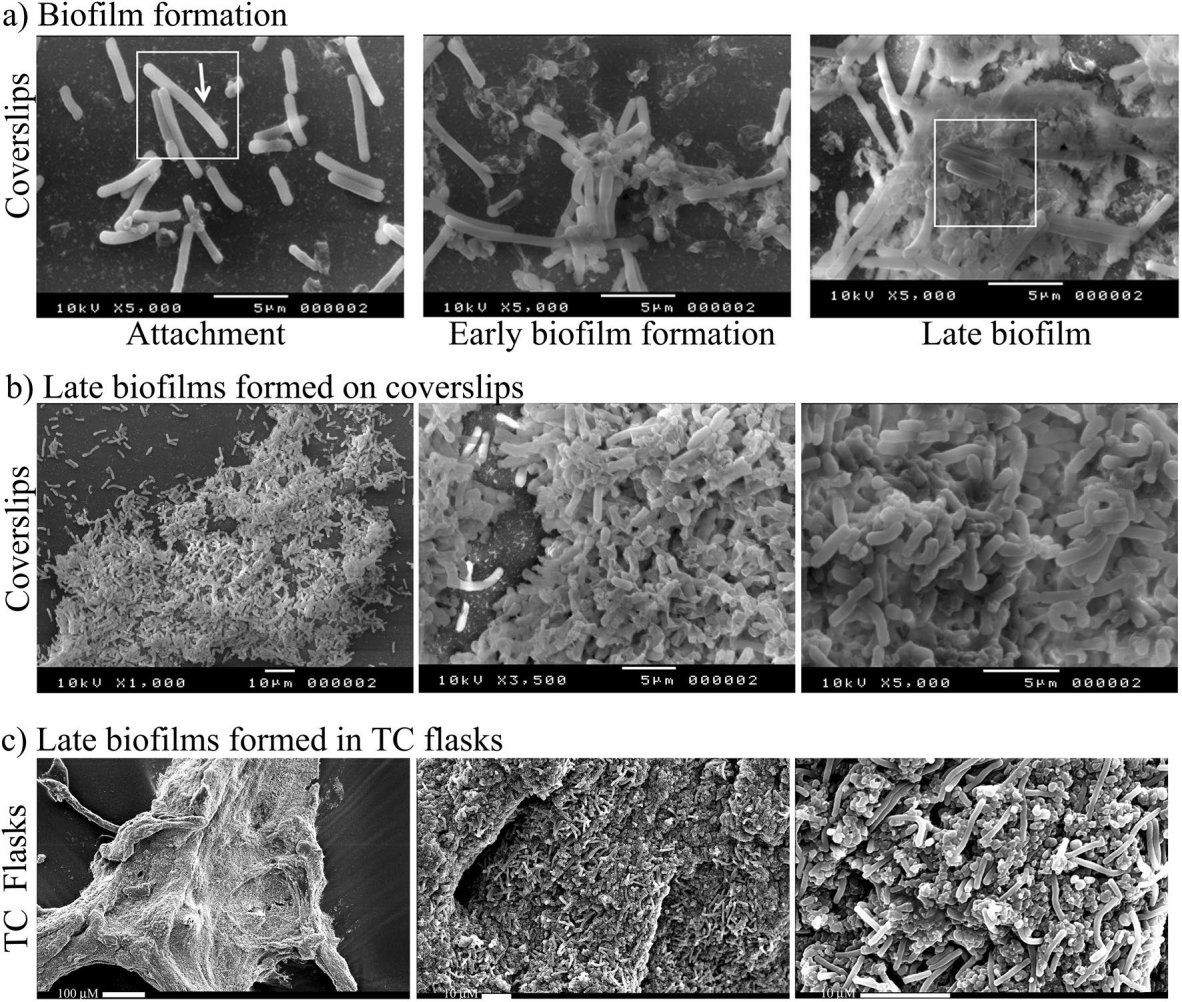
A schematic and SEM visualisation of biofilm formation of *C. difficile* strain 630. (**a**) visualization of three stages of biofilm formation grown on coverslips in 24-well plates for 16 h (attachment), 24 h (early biofilm) and 72 h (late biofilms) prior to visualization. Surface attached appendages are indicated with an arrow, within the white box. White boxes indicate high magnification images present in supplementary data (Supplementary Fig. S1 online). Scale bar 5 µm. (**b**,**c**) Comparison of late biofilms formed on coverslips in 24 well plates and in tissue culture flasks, fixed and transferred to a stub for imaging. (**b**) Scale bar 10 µm (×1000), 5 µm (×3500 and ×5000), (**c**) scale bar 100 µm (×150), 10 µm (×1000 and ×3000).

*Clostridioides difficile* strain 630, a ribotype 012 (RT012) was grown on thermanox coverslips in BHIS broth under conditions that allowed biofilm formation. Using scanning electron microscopy (SEM), we show three distinct stages of biofilm formation: initial attachment of bacteria to an abiotic surface via bacterial appendages, through to early biofilm formation, where we observe formation of a matrix, culminating at late biofilm formation, in which the bacteria appear to be encased within an extracellular matrix (Fig. 1 a and Supplementary Fig. S1 online). Late biofilms formed in static TC flasks, were detached after a 72 h incubation from the abiotic surface (bottom) by gentle agitation, fixed and transferred onto coverslips for SEM imaging (Fig. 1c). The morphology and structure of biofilms formed in static TC flasks (Fig. 1c) is similar to biofilms formed directly on coverslips in 24-well plates (Fig. 1b).

#### Structure of the biofilm matrix

Visualization by confocal microscopy of *C. difficile* biofilms produced by strains 630 and R20291 with a FilmTracer Live-Dead stain, revealed the presence of viable and non-viable cells, encased within an extracellular matrix (Supplementary Fig. S2a online). Nucleic acid and less abundant protein components within the extracellular matrix were positively stained with Acridine Orange, DAPI and SYPRO-Ruby Matrix stain respectively (Supplementary Fig. S2a online). Late biofilms were disrupted with the addition of DNase and visualized by DAPI staining (Supplementary Fig. S2b online). The concentration of eDNA alone and total DNA (intracellular and eDNA) was significantly less in biofilms formed by R20291 (12.7 ± 0.7 mg/biofilm) compared to 630 (18.5 ± 1.6 mg/biofilm) (Supplementary Fig. S2c online).

#### Composition of *C. difficile* biofilms from representative strains of five lineages

**Figure 2:**
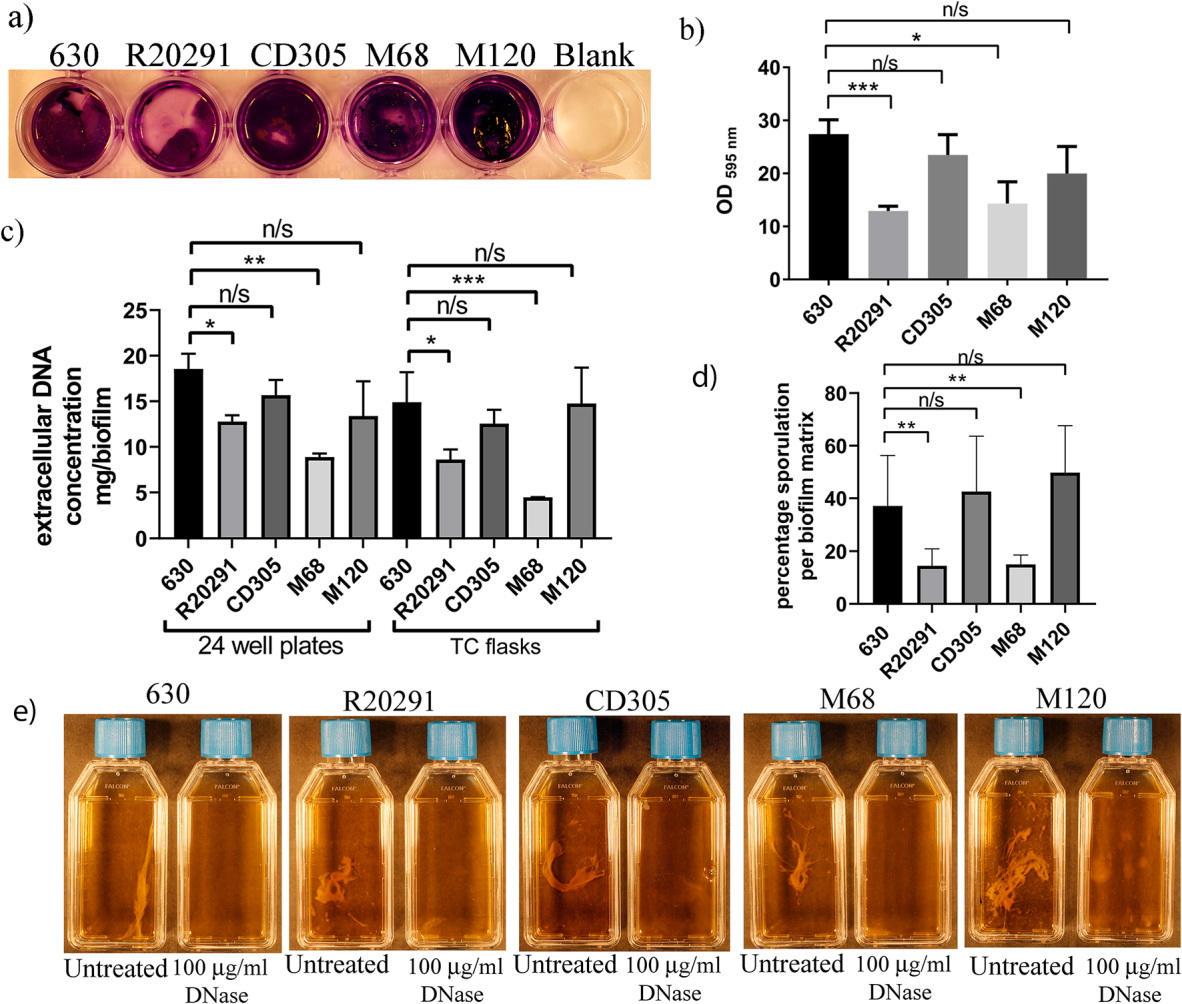
Biofilm formation and eDNA concentrations of representative strains from each of the five *C. difficile* lineages. (**a**) Biofilm biomass determined by crystal violet assays performed in 24-well plates. (**b**) Quantification of biofilm biomass by methanol extraction of crystal violet assays performed in 24-well plates. Crystal violet assays are representative of six independent biological replicates. (**c**) Concentration of eDNA (*C. difficile* cells and spores were removed prior to eDNA quantification by filtering the vortexed disrupted biofilm samples) from duplicate biofilms produced in 24-well plates compared to TC flasks in a representative strain of all five *C. difficile* lineages: 630 (RT012), R20291 (RT027), CD305 (RT023), M68 (RT017) and M120 (RT078). (**d**) Percentage sporulation in the biofilm matrix produced in TC flasks for each strain in biological triplicates. (**e**) Duplicate late biofilm cultures were grown in tissue culture flasks, then detached by gentle agitation from the bottom of the tissue culture flask. One was left untreated and the other treated with 100 µg/mL DNase for 15 min to disrupt the biofilm matrix. Images of the tissue culture flasks were taken with a Cannon 600D SLR (mounted on a copy stand with lighting unit (Kaiser RS2) with a 50 mm prime lens). This was undertaken for *C. difficile* strains from all five lineages: 630 (RT012), R20291 (RT027), M120 (RT078), M68 (RT017) and CD305 (RT023). All error bars are SD. Statistical differences were assessed using linear regression analysis and significant differences are indicated *p < 0.05, **p < 0.01, ***p < 0.001.

Representatives from all five *C. difficile* lineages produce robust biofilms, however, R20291 (RT027) and M68 (RT017) form biofilms with significantly less biomass than 630 (RT012), CD305 (RT023) and M120 (RT078) (Fig. 2a,b). Biofilms from all five *C. difficile* lineages contain eDNA in their matrix (Fig. 2c), however, the concentration of eDNA correlated directly with biofilm biomass: strains R20291 (RT027) and M68 (RT017) contain significantly less eDNA in their matrix than 630 (RT012) (p < 0.05 and p < 0.01 respectively) (Fig. 2c; Table 2). Biofilms produced by all five *C. difficile* lineages in TC flasks were degraded by the addition of exogenous DNase (Fig. 2e), indicating that eDNA is an integral part of the biofilm matrix for all five lineages. We detected and quantified vegetative cells and spores encased within the biofilm matrix, compared to the planktonic phase from all five lineages (Supplementary Fig. S3 online). We observed a similar sporulation frequency in the biofilm matrix of strains CD305 (RT023) (42.6% ± 12.1) and M120 (RT078) (49.7% ± 7.3) compared to 630 (RT012) (37.1% ± 9.6) (Fig. 2d). Conversely, we observed a significant reduction in the percentage sporulation within the biofilm matrix of strains R20291 (RT027) (14.5% ± 3.7) and M68 (RT017 (Fig. 2d) (15.0% ± 1.43), compared to 630 (*p* < 0.01), which is linked to reduced eDNA concentration within the biofilm matrix (*p* < 0.01) and decreased biofilm biomass (*p* < 0.01) (Fig. 2). Overall, we observed a significant increase in spore titre (*p* < 0.01) within the biofilm matrix compared to the planktonic phase (supernatant) for all strains with the exception of CD305 (Supplementary Fig. S3 online), where the sporulation frequency is similar in both the planktonic culture and biofilm. All five *C. difficile* lineages exhibit a positive correlation between biofilm biomass, eDNA concentration and percentage sporulation within the biofilm matrix (Fig. 2).

### The role of cell surface proteins in early biofilm formation

*Clostridioides difficile* encodes a number of cell surface proteins, either non-covalently anchored to the cell wall or covalently anchored to peptidoglycan via sortase SrtB^48–51^. A number of cell wall associated proteins are regulated by the secondary messenger c-di-GMP. We therefore investigated the impact of c-di-GMP and sortase substrates of *C. difficile* on biofilm formation.

#### The effect of the secondary messenger c-di-GMP on attachment and biofilm formation

**Figure 3:**
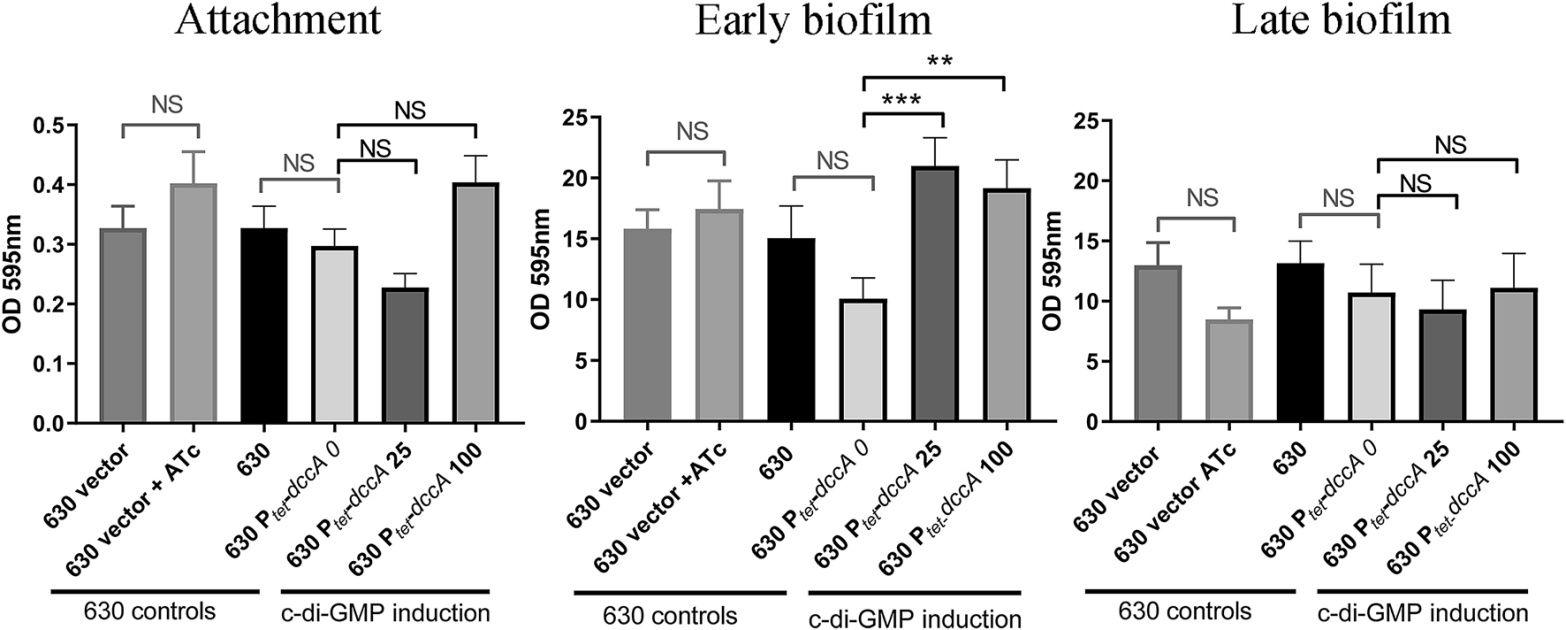
The effect of the secondary messenger c-di-GMP on biofilm formation for *C. difficile* strain 630. In-vitro attachment, early biofilm and late biofilm formation in 24 well plates were quantified using crystal violet assays with strains of *C. difficile* 630 containing an empty plasmid (630 vector), or with a plasmid-based diguanylate cyclase (*dccA*) under control of a *P*_*tet*_ (inducible) promoter. These were compared to a 630 control strain containing a plasmid in the presence of 100 ng/mL anhydrotetracycline (630 vector + ATc). Biofilm and attachment assays were performed with a minimum of biological quadruplicate. The concentration of ATc used to induce *dccA* is listed in the graphs (0, 25 or 100 ng/mL), strain labelled 630P_*tet*_* dccA* 0 is an uninduced vector control. Error bars are SD. Significant differences are calculated using linear regression analysis compared to the uninduced control strain 630P_*tet*_* dccA* 0, **p < 0.01, ***p < 0.001.

C-di-GMP has been shown to promote biofilm formation^42,44,53^*.* In order to investigate whether elevated levels of c-di-GMP altered attachment, early biofilm (24 h) or late biofilm formation (72 h), we used *C. difficile* strain 630 harbouring a plasmid encoding the diguanylate cyclase gene *dccA* under the transcriptional control of an anhydrotetracycline (ATc) inducible promoter (*P*_*tet*_)^49^. In line with Purcell et al.^44,57^, we observed a significant increase in early biofilm formation (Fig. 3) with induction of *dccA* (*p* < 0.001 at 25 ng/mL and *p* < 0.01 at100 ng/mL ATc), which elevates c-di-GMP. There was no significant difference in the effect of c-di-GMP on late biofilm formation or initial attachment and no significant modulatory effect of exogenous anhydrotetracycline added to control cells, at any stage of biofilm formation (Fig. 3). This indicates that the significant increases in biofilm biomass observed during early biofilm formation were due to c-di-GMP induction.

#### The role of PPEP-1 cleaved cell surface proteins in early biofilm formation

**Figure 4:**
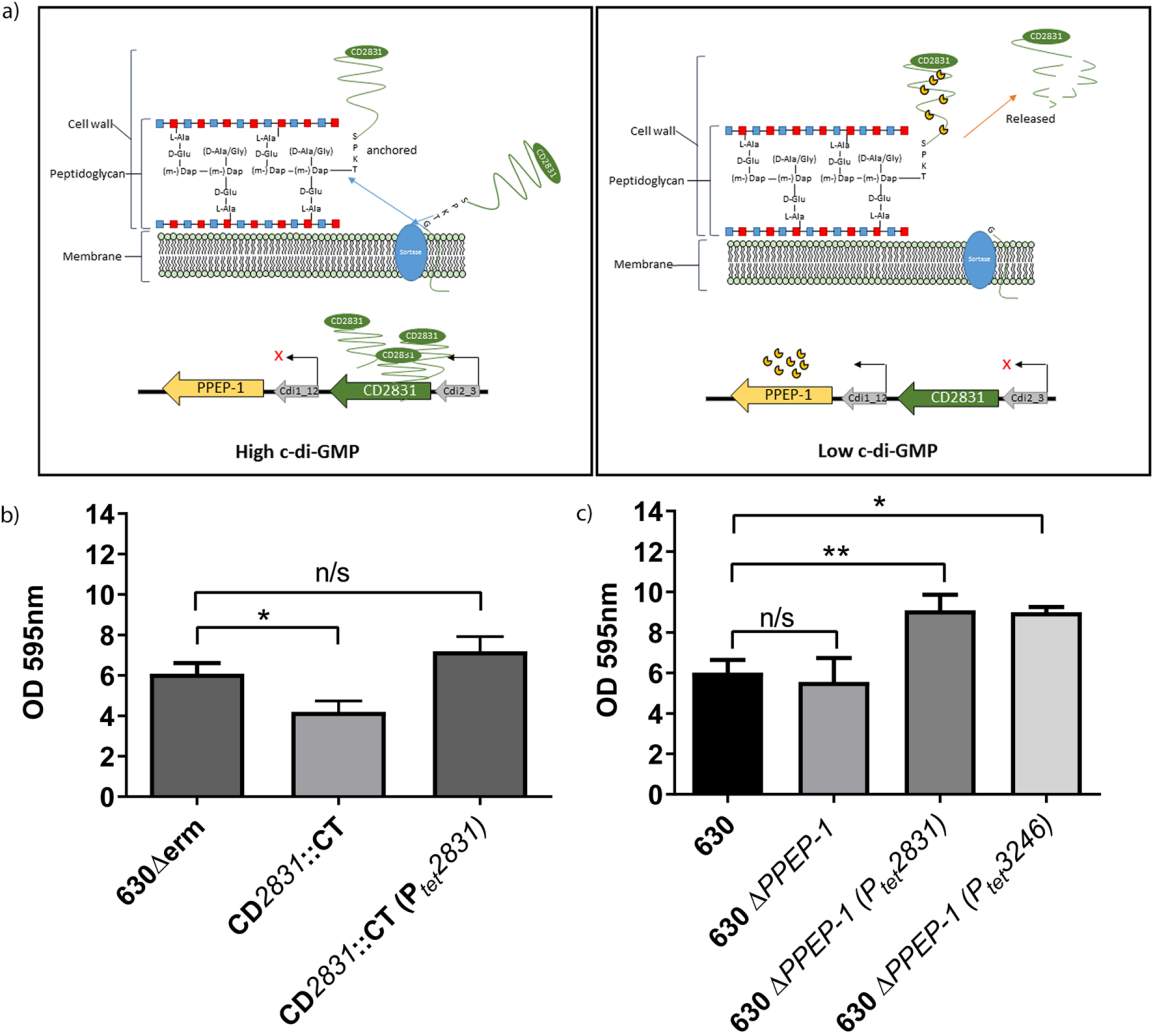
The effect of c-di-GMP regulated cell wall proteins on biofilm formation for *C. difficile* strain 630. (**a**) Schematic showing the sortase-dependent anchoring of CD2831 to the peptidoglycan of *C. difficile*. Under high levels of c-di-GMP, *CD2831* is expressed by the permissive interaction of c-di-GMP with the upstream type II riboswitch (Cdi2_3). The protease *PPEP-1* is repressed under high levels of c-di-GMP due to the upstream type I riboswitch (Cdi1_12). Under low levels of c-di-GMP, expression of *CD2831* is repressed and *PPEP-1* is upregulated, resulting in the cleavage of CD2831 (and CD3246) from the peptidoglycan and release into the supernatant. This scheme is based on published data^49,55,56^. (**b**) Crystal violet quantification of early biofilm formation produced in 24 well plates with an insertional inactivation mutant of *CD2831* compared to its wildtype and inducible *CD2831*complement under control of anhydrotetracycline (ATc). (**c**) Crystal violet quantification of early biofilm formation produced in 24 well plates with overexpression of *CD2831* and *CD3246* under the control of an ATc inducible promoter, in an in-frame deletion mutant of the protease *PPEP-1*. Crystal violet assays were performed with a minimum of 6 biological replicates. Error bars are SD. Significant differences are calculated using linear regression analysis compared to all strains against its respective parent strain 630Δ*erm* or 630, *p < 0.05, **p < 0.01.

**Figure 5:**
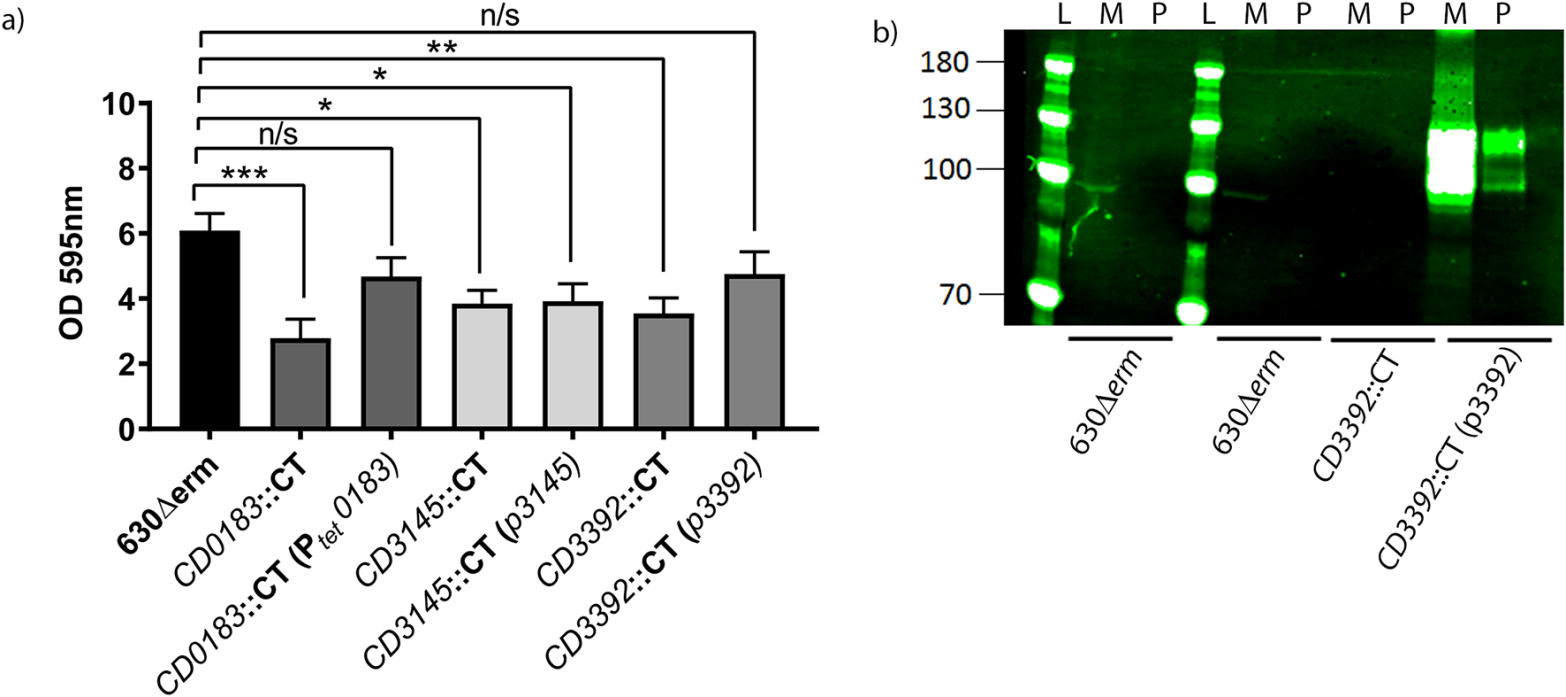
Cell wall proteins involved in biofilm formation. (**a**) Insertional inactivation mutants of non-c-di-GMP regulated proteins CD0183, CD3145 and CD3392 were created in *C. difficile* strain 630Δ*erm*. Mutants were complemented by introduction of plasmids expressing the wild type gene. These mutants and their complements were assessed for their ability to form early biofilms (24 h) on an abiotic surface in vitro compared to a wild-type control. Crystal violet assays were performed with a minimum of 6 biological replicates. All error bars are SD. Statistical differences were assessed using linear regression to compared to all strains against their respective parent strain 630Δ*erm*, *p < 0.05, **p < 0.01, ***p < 0.001. (**b**) Western blot using anti CD3392 antibodies of the matrix (M) and planktonic fractions (P) of a late biofilm, from strains 630Δ*erm*, *CD3392*::CT and CD3392 complement (*CD3392*::CT (p*CD3392*)). The biofilm matrix was detached from the bottom of a TC flask, disrupted by the addition of DNase (100 µg/mL), then loaded onto an SDS-PAGE gel alongside a protein ladder (L) for analysis by Western blot.

Surface exposure of the sorted proteins CD2831 and CD3246 is tightly controlled by a c-di-GMP dependent pathway, via a type II riboswitch, summarized in Fig. 4A. There is an inverse transcriptional regulation of *CD2831* and *CD3246* with the metalloprotease PPEP-1, which is under the control of a c-di-GMP dependent type I riboswitch (Fig. 4A). Elevated c-di-GMP increases expression of surface localized CD2831^49^ and CD3246 proteins^54,55^. Gene inactivation of *CD2831* (Supplementary Fig. S4 online) significantly reduced early biofilm formation (*p* < 0.05, Fig. 5B), which is complemented *in trans* with the addition of *CD2831* under the control of an anhydrotetracycline (ATc) inducible promoter (Fig. 4B). In-frame deletion of *PPEP-1* does not significantly alter early biofilm formation (Fig. 4C), however, over-expression of *CD2831* or *CD3246* in a *PPEP-1* mutant significantly enhances early biofilm formation (*p* < 0.01, *p* < 0.05 respectively, Fig. 4C). This suggests that CD2831 and CD3246 are important in early biofilm formation in the presence of high levels of c-di-GMP, when expression of the peptidase PPEP-1 would be low, as this would result in CD2831 and CD3246 remaining attached to the cell wall of *C. difficile*.

#### The role of additional cell surface proteins in early biofilm formation

Using gene inactivation mutagenesis, we studied the roles of other cell wall proteins in biofilm formation. CD3392 is a sortase substrate and is covalently anchored to the cell wall^52,54,55^. CD0183 contains the canonical (S/P)PXTG motif, but is not processed by sortase and is partially bound to the cell wall, presumably by non-covalent interactions^49,50^. CD3145 (CbpA) carries an LPXTG-like cell wall anchoring domain, but is not processed by sortase^52^, however it is expressed on the cell surface and binds collagens I and V with high affinity^58^.

Mutation of *CD3392* or *CD0183* significantly reduces early biofilm formation (*p* < 0.01, Fig. 5a), which is complemented by *CD3392* or *CD0183* expression, *in trans* (Fig. 5a). Mutation of CD3145 (CbpA), also significantly reduced biofilm formation, however, this phenotype was not successfully complemented with expression of *cbpA in trans* (Fig. 5a).

**Figure 6:**
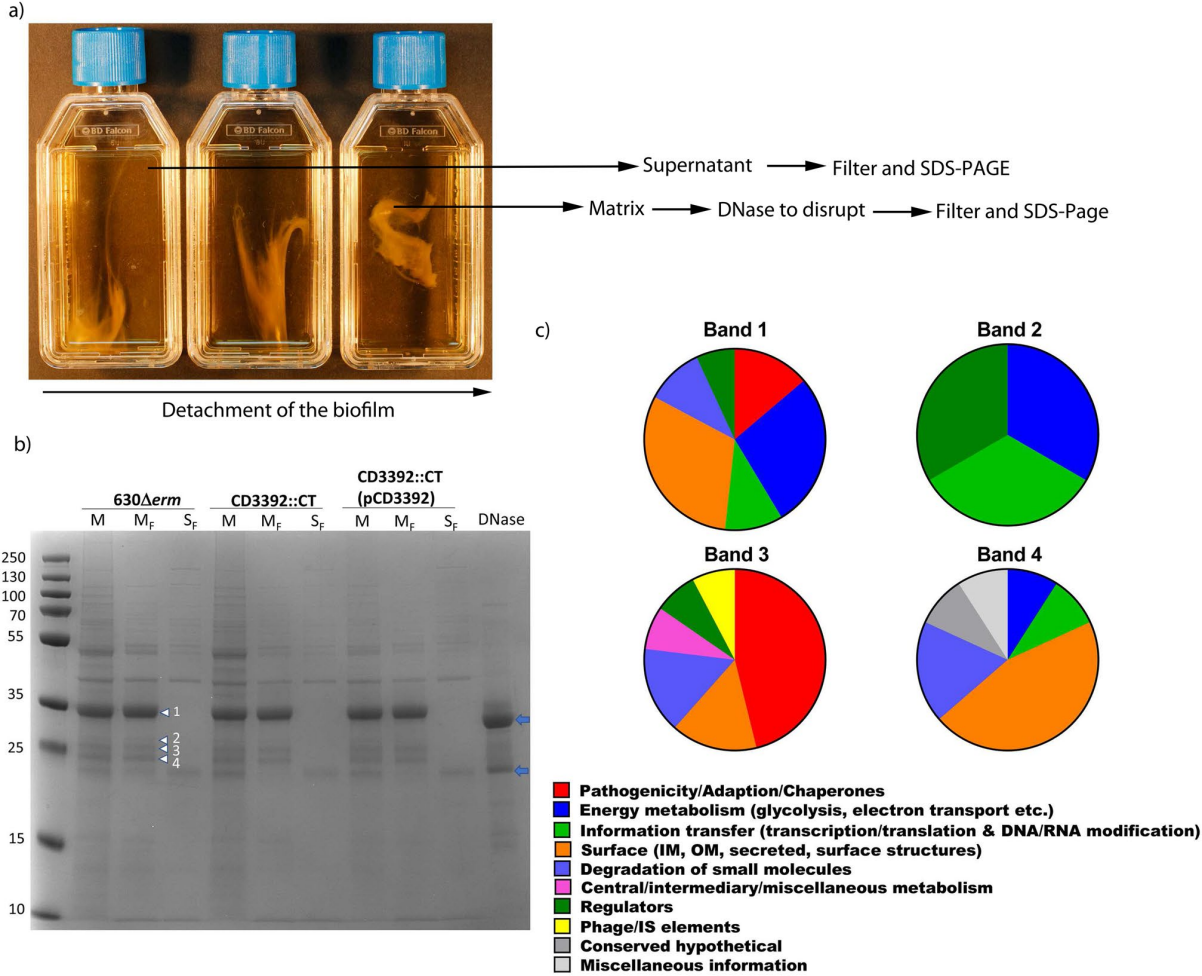
Analysis of the biofilm matrix and planktonic fractions. (**a**) Separation of the biofilm matrix and supernatant (planktonic fraction). After the biofilm matrix was detached from the bottom of the TC flask, the biofilm matrix was disrupted by the addition of DNase (100 µg/mL). (**b**) SDS-PAGE electrophoresis of the Biofilm matrix after DNase processing (M), filtered biofilm matrix (M_F_), compared to the filtered planktonic fraction (S_F_), and DNase control. The arrows on the left label the bands (1–4 top to bottom) sent for LC–MS/MS from strain 630∆*erm*, the blue arrows indicate the major and minor DNase band within the DNase control lanes. (**c**) Protein classification of the bands 1–4 presented in pie charts, with the percentage normalized total spectra. Colour codes indicate different functional classifications in accordance with the Riley classification.

We then asked whether CD3392 could be detected within the biofilm matrix (Fig. 5b). We separated the biofilm matrix from the planktonic fraction (culture supernatant) of biofilms grown in TC flasks (Fig. 6a). The biofilm matrix fraction was disrupted by DNase treatment and both the matrix and supernatant fractions were filtered to remove *C. difficile* cells prior to SDS-PAGE and western blotting. We identified CD3392 predominantly in the matrix fraction of *C. difficile* cultures, with none detectable in the planktonic fraction (Fig. 5b). CD3392 was not detected in the matrix from a mutant strain, but was present in large amounts in the matrix from a complemented strain, suggesting CD3392 may form part of the biofilm matrix.

#### Detection of matrix proteins

To identify further proteins in the matrix, biofilms were produced in TC flasks and the biofilm matrix fraction was separated from the planktonic fraction (culture supernatant) (Fig. 6a). The biofilm matrix fraction was disrupted by (1) vortexing prior to quantification by BCA assay, or (2) by DNase treatment prior to SDS-PAGE analysis. After disruption of the biofilm matrix, aliquots from both the matrix and supernatant fractions were filtered to remove *C. difficile* cells before analysis was undertaken. We performed LC–MS/MS on the four main bands identified by SDS-PAGE as unique to the filtered biofilm matrix from *C. difficile* strain 630∆*erm* (Fig. 6b). These four bands analysed were also present in the biofilm matrix of the *CD3392* mutant and complement strains, indicating their potential importance in biofilm formation (Fig. 6). Analysis of these bands indicate the presence of multiple *C. difficile* proteins: 12 proteins in band 1, 5 proteins in band 2, 13 proteins in band 3 and 10 proteins in band 4 (Fig. 6c). The predominant band present in the matrix fraction (1) was a combination of the low molecular weight (LMW) portion of the major surface layer protein SlpA, with intracellular proteins, which include those involved in butyrate fermentation (Hbd, Buk and ThlA1), oxygen stress tolerance (Rbr), amino acid degradation (GluD) and the utilization of glutamate and proline by Stickland fermentation (PrdA) (Fig. 6; Supplemenatry Table S1). Alongside these *C. difficile* proteins, bovine DNase used to disrupt the biofilm was also detected in Band 1. The other three bands unique to the biofilm matrix (2, 3 and 4) were composed of a combination of *C. difficile* proteins, including; cell surface proteins (FbpA, Cwp19, Cwp6, CD1131, SlpA), pathogenicity and adaption proteins (PotA and ModA (ABC transporters)), Rbr (Rubrerythrin) and DnaK (chaperone), a phage protein, and proteins involved in amino acid metabolism (proline), fermentation (butyrate) and phosphoenolpyruvate-dependent sugar phosphotransferase system (PTS) (Supplemenatry Table S1; Fig. 6).

### Biofilm disassembly and resistance to vancomycin

**Figure 7:**
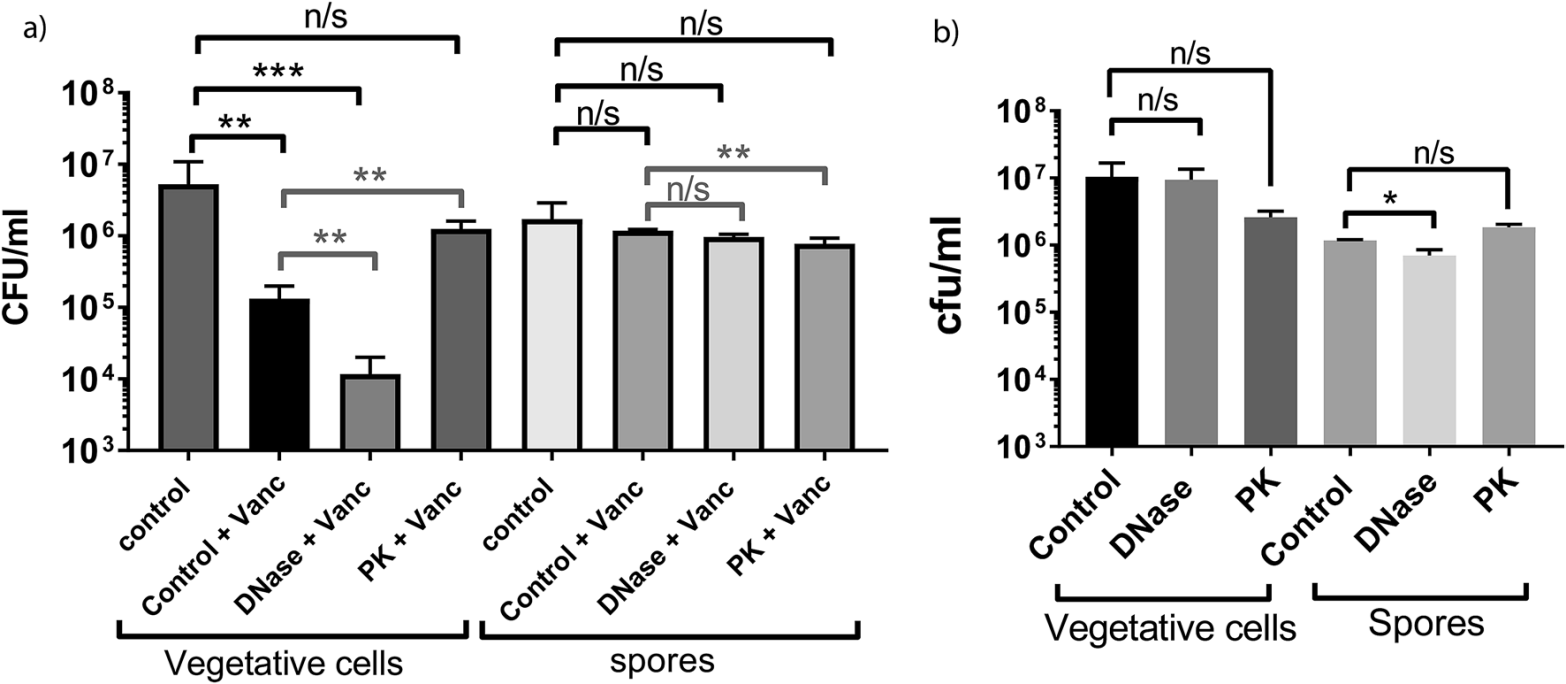
The effect of vancomycin on CFU counts in disrupted and intact *C. difficile* strain 630 biofilms. (**a**) The effect of vancomycin treatment alone or in combination with DNase and Proteinase K on both vegetative cells and spores within an intact and degraded biofilm was undertaken in 3-day old biofilms, compared to untreated 3-day old biofilms. (**b**) A comparison between the effects of either DNase or proteinase K on the viability of vegetative cells and spores within a biofilm. The biofilms were detached from the bottom of the flask by gentle agitation and pre-treated with either vancomycin, or vancomycin supplemented with recombinant DNase or Proteinase K, compared to an untreated intact biofilm. Total counts and spores were enumerated and differentiated by heat inactivation of spore samples, thus killing the vegetative cells, enumerating spores alone. Experiments were undertaken with a minimum of 3 biological replicates. The data was analysed in Excel and GraphPad Prism 7.0, and error bars represent SD. Statistical analysis to determine the effect of vancomycin on disrupted and intact biofilm was performed using linear regression, **p < 0.01 (grey) and the effect of vancomycin and vancomycin combined with DNase and Proteinase K compared to untreated biofilms *p < 0.05, **p < 0.01, ***p < 0.001 (black).

DNase both inhibited biofilm production and disassembled preformed biofilm in TC flasks and on coverslips, whereas Proteinase K partially disrupted preformed biofilms. In contrast, bovine serum albumin had no measurable effect on biofilm initiation and RNase did not influence biofilm integrity (Supplementary Fig. S5 online). Therefore, we assessed the effects of the biofilm dispersal agents, DNase and Proteinase K on the vulnerability of vegetative cells and spores to the antibiotic vancomycin (Fig. 7), by comparing the viable counts of vegetative cells and spores within intact and disrupted biofilms in the presence of vancomycin. We observed that vancomycin alone significantly reduced the viable vegetative cell count in intact biofilms (*p* < 0.01 COV = − 6.243) to 7.7% of the untreated level, whereas disruption with DNase (*p* < 0.01 COV = − 10.805) combined with vancomycin treatment reduced the vegetative cell count to 0.68% of the untreated control. However, treatment with proteinase K in combination with vancomycin treatment only reduced the viable vegetative cell counts to 72.8% of an untreated biofilm (*p* = 0.916 COV = − 0.309) (Table 2), which was less effective than the addition of vancomycin alone, suggesting that proteinase K may affect the structural integrity of the glycopeptide antibiotic vancomycin, thus reducing its potency. This indicates that the combined effect of DNase and vancomycin produced the most significant reduction in viable vegetative cell counts. As expected, vancomycin had no effect on spore viability irrespective of biofilm disruption, compared to the untreated biofilms (Fig. 7a; Table 2). The addition of DNase alone had no effect on vegetative cells (*p* = 0.989, COV = 0.010) viability, however DNase had a small but significant effect on spore viability in the biofilm matrix (*p* < 0.05 COV = − 0.568) (Fig. 7b) and the planktonic fraction *p* < 0.01 (Supplementary Fig. S6 online). The addition of proteinase K alone had no effect on the viability of vegetative cells (*p* = 0.897 COV = − 0.086) or spores (p = 0.109, COV = 0.437) compared to the untreated biofilms (Fig. 7b). However, proteinase K decreased the relative proportion of spores (p < 0.05, COV = − 0.494) in the presence of vancomycin (Fig. 7a; Table 2) compared to the vancomycin treated biofilms, suggesting that proteinase K reduced the spores’ viability only in the presence of vancomycin. This adds additional evidence to the protective nature of the biofilm and the efficacy of DNase to disrupt the biofilm and reduce spore viability. Additionally, when used in combination with a current first line therapy DNase promotes the efficacy of vancomycin to reduce the viability of vegetative cells.

## Discussion

In this study, we identify several factors involved in biofilm formation in all five *C. difficile* lineages, as well as exogenous factors involved in biofilm disassembly, which enhance the efficacy of vancomycin, a first line anti *C. difficile* treatment (summarized in Supplementary Fig. S7 online). A greater understanding of biofilm formation, dispersal and inhibition in other pathogenic bacteria has reduced disease burden and prevented relapse of infections^59^. Many patients suffer from long lasting chronic infections, driven by bacteria persisting in biofilms or other protected niches, impenetrable or intrinsically resistant to antibiotic therapy^7,60^. The current crisis in widespread antimicrobial resistance has raised fears over an era of untreatable infections^61^.

The purpose of a biofilm matrix is aligned within and between bacterial species to protect the cells encased therein from environmental stresses, antibiotics and the immune response^6^. Interestingly, several bacterial species modulate their biofilm matrix composition between (1) exopolysaccharide, (2) extracellular and cell surface proteins and (3) extracellular DNA (eDNA)^13^: The human pathogens *S. aureus* and *P. aeruginosa* produce multiple distinct biofilm matrix compositions^19,62^. In *S. aureus*, biofilm composition can be proteinaceous (cell surface proteins and teichoic acid) combined with eDNA or alternatively a specifically synthesized exopolysaccharide [β-1,6-linked *N*-acetylglucosamine (PIA/PNAG)]^45,63^, yet the clinical importance of these two variants is not completely understood^19^. Combinations of eDNA, proteins and polysaccharides have been identified surrounding adherent colonies of *C. difficile*, although the extent to which they contribute to biofilm formation and integrity remains to be determined^24,25^. In this study, we show that clinical strains representing each of the five *C. difficile* lineages RT012 (630), RT027 (R20291), RT023 (CD305), RT017 (M68) and RT078 (M120) produce biofilms on an abiotic surface, comprised of vegetative cells and spores encased within an EPS matrix comprised mainly of eDNA combined with intracellular and cell surface associated proteins.

In *P. aeruginosa*, temporal and genetic control of eDNA release by cell lysis is essential in initial development of biofilm formation^18,20^ and for many pathogenic bacteria, including *P. aeruginosa*, *Enterococcus faecalis* and *L. monocytogenes* DNA is a major component of the biofilm matrix^18,21,64,65^. In this study, we show that biofilm biomass positively correlates with eDNA concentrations within the biofilm matrix. Representatives of three *C. difficile* lineages, RT012(630), RT023(CD305) and RT078(M120) form larger more robust biofilms with a higher eDNA concentration and an increased sporulation frequency within the biofilm matrix, compared to representatives of the other two *C. difficile* lineages, RT027 (R20291) and RT017(M68). We show that without DNA, a major component of the biofilm matrix in *C. difficile*, initial biofilm formation is severely inhibited, and late biofilms are disassembled in all five *C. difficile* lineages. Our data complements a study from Dubois et al., who identified eDNA as a component of *C. difficile* biofilms formed by strain 630∆*erm* in the presence of the secondary bile salt deoxycholate (DOC), which is present in the human gut and toxic to vegetative cells^29^. The structural importance of eDNA should not be underestimated, in other species eDNA aids bacterial adhesion, stabilizes the biofilm structure and contributes to antimicrobial resistance, by chelating cations and restricting diffusion of cationic antimicrobials^66^. Environmental conditions can trigger alterations in the abundance of exopolysaccharides and eDNA within *S. aureus* biofilms, however, even small quantities of eDNA are essential for biofilm integrity^67^. Herein, we demonstrate that although there are variations in eDNA concentration within the biofilm matrix of different *C. difficile* lineages, eDNA is a key factor in development and structural integrity of *C. difficile* biofilms across all five lineages.

We detected a significantly higher spore titre in the biofilm matrix compared to the planktonic phase for four of the five lineages. *C. difficile* sporulation is linked to persistence of CDI^68,69^ and is mediated by the sporulation master regulator *spo0A*, inactivation of which results in a significant reduction in biofilm biomass^23,25,29^, indicating a key role for Spo0A in biofilm formation. Biofilms enhance persistence for many chronic bacterial infections, providing protection from environmental stresses and antibiotics^60^. Therefore, we hypothesise that Spo0A may support *C. difficile* biofilm formation and persistence, by inducing sporulation and consequently cell lysis during stationary phase, resulting in a release of eDNA, which can be incorporated into the biofilm matrix. Vegetative cells and spore encased within these protective biofilms could be released to re-seed the gut and facilitate relapse of CDI.

Biofilm architecture is a key factor of recalcitrance (resistance to antibiotics), which affects drug diffusion, as well as initiation of the starvation and SOS response, which all contribute to biofilm stability^7^. Biofilms produced by different bacterial species can be artificially disrupted by the addition of exogenous polyamines, DNase, d-amino acids or alginate lyase depending of the structure and composition of the biofilm matrix^18,71–73^. To eradicate chronic infections combinatorial approaches that induce biofilm dispersal via enzymatic degradation (DNase, Dispersin A or glucanhydrolases) with current antibiotic therapy^7^ enhances eradication of pathogens such as *Streptococcus mutans* and *P. aeruginosa in* cystic fibrosis patients^75^. Herein, we show that DNase successfully disassembles biofilms produced by all five *C. difficile* lineages and that degradation of eDNA within the biofilm by DNase, increases the effectiveness of vancomycin from 92.3 to 99.3% at reducing vegetative cell titre. We show DNase reduced the viability of *C. difficile* spores in both the biofilm matrix and planktonic fraction, measured by germination, which aligns with a study in *B. subtilis*, showing a combination of lysozyme and DNase damaged spore coat proteins affecting viability and germination^76^. Dapa et al. observed a decrease in biofilm integrity in preformed biofilms with both proteinase K and DNase, with extended incubation^25^ and Dubois et al., found both DNase and proteinase K dispersed *C. difficile* biofilms induced in the presence of the secondary bile salt deoxycholate^29^. However, in our system, proteinase K only marginally reduced biofilm biomass, this potentially results from media differences, as Dubois et al., used BHISG growth media, known to preferentially promote exopolysaccharide production and reduce eDNA within a *S. aureus* biofilm^67^. More importantly, we show that proteinase K reduced the bactericidal effect of vancomycin on *C. difficile*, indicating that proteinase K should not be used synergistically with vancomycin. This highlights the merits of a dual approach for reducing *C. difficile* biofilm recalcitrance, in which DNase reduces both spore germination and enhances the efficacy of vancomycin.

In many organisms, particularly Gram-negative bacteria, elevated levels of the secondary messenger c-di-GMP promote biofilm formation by regulating many cellular systems, including biosynthesis of matrix polysaccharides in *P. aeruginosa*^59,62^*, E. coli*^77^ and *S. aureus*^45^*,* whereas low level c-di-GMP inhibits biofilm formation^54,78^. Sub-inhibitory concentrations of certain antibiotics trigger an increase of c-di-GMP to promote biofilm formation in *E. coli* and *P. aeruginosa*^62,77^. *C. difficile* encodes multiple enzymes involved in the synthesis (diguanylate cyclases) and degradation (phosphodiesterases) of c-di-GMP^79^. In *C. difficile*, c-di-GMP inversely regulates aggregation and cell motility^57^, by modulating expression of the *flgB* flagella operon^43^ and the type IV pili cluster^80^*.* Herein, we show that c-di-GMP enhances early biofilm formation, corroborating observations from Soutourina et al*.*^53^ and Purcell et al.^44,57^, however, c-di-GMP has little effect on attachment and late biofilm formation. Some cell wall anchored proteins in *C. difficile*, including CD2831 and CD3246 are regulated via a c-di-GMP dependent pathway, which increases transcription via a type II dependent riboswitch^54^. Under elevated c-di-GMP, CD2381 and CD3246 are covalently attached to peptidoglycan in the cell wall of *C. difficile* via a sortase enzyme (SrtB)^49^. Conversely, under low level c-di-GMP, *PPEP-1*, a metalloprotease (CD2830/ZmpI) controlled by type I dependent riboswitch is upregulated and cleaves CD2831 and CD3246 from the cell wall^54^. We observed that the cell wall protein CD2831 enhances early biofilm formation in *C. difficile* and overexpression of *CD2831* or *CD3246* in a *PPEP-1* deletion background mimics an elevation of c-di-GMP levels, which results in significantly increased biofilm formation. Similar to our observations, surface associated cell wall proteins regulated by sortase in *S. aureus* are linked to biofilm formation^47^. We speculate that increased biofilm formation under elevated c-di-GMP (*in trans*) in early biofilm formation could result from reduced cleavage of CD2831 and CD3246 from the cell wall, as well as promoting expression of c-di-GMP dependent genes, these include cell wall genes, PTS systems and ABC transporters, that were identified by McKee et al*. *as directly or indirectly regulated by c-di-GMP riboswitches^54^*.* In line with our results, Arato et al. showed a contribution of CD2831 in biofilm formation^81^, whereas Poquet et al.^28^ did not observe a difference in biofilm formation upon deletion of *CD2831*. However, Poquet et al., used BHISG media for biofilm formation, which as discussed, triggers environmental biofilm variants in *S. aureus*, with increased exopolysaccharide formation and reduced eDNA^67^. In some species, environmental conditions trigger a biofilm matrix preferentially composed of cell surface proteins alongside eDNA^47,82,83^. In addition to media, differences in biofilm composition in *C. difficile* observed between laboratories may be a result of variation in intrinsic c-di-GMP levels, which regulate the cleavage of CD2831 from the peptidoglycan via PPEP-1. To address this, we overexpressed *CD2831* and *CD3246* in a *PPEP-1* deletion strain and showed a significant increase in biofilm formation. We suggest that temporal production of c-di-GMP positively modulates early biofilm formation, rather than increasing biomass of an established biofilm by modulating attachment of cell wall proteins to peptidoglycan, alongside upregulating PTS systems and ABC transporters identified by McKee et al. to modulate metabolism within a biofilm and downregulate motility, which favors a sessile lifestyle (Supplementary Fig. S7 online). Alongside the c-di-GMP regulated cell surface proteins, we also identified non-c-di-GMP regulated cell surface proteins which contributed to early biofilm formation in *C. difficile*, some of which are covalently anchored to peptidoglycan via the sortase (StrB)^49,50,52^. We identified that deletion of the cell surface proteins CD0183; a putative cell wall hydrolase, CbpA; a collagen binding protein and CD3392; a predicted collagen binding protein, significantly reduced the biomass of early biofilms. Several sortase substrates of *S. aureus*, including FnbpA, FnbpB, ClfA and CflB, are known to promote biofilm formation^84^. Interestingly, methicillin resistant *S. aureus* (MRSA) forms biofilms with predominantly cell surface proteins and eDNA, whereas methicillin sensitive *S. aureus* (MSSA) forms biofilms with the PIA exopolysaccharide^47^. This highlights the role for a combination of c-di-GMP dependent and c-di-GMP independent cell surface proteins and eDNA within a biofilm matrix.

We, like Semenyuk et al.^85^ were unable to identify a specific highly produced exopolysaccharide in the *C. difficile* biofilm matrix. We observed the presence of c-di-GMP dependent and c-di-GMP independent cell wall proteins, PTS and ABC transporters, and intracellular proteins within the biofilm matrix of *C. difficile*, which contribute to the biofilm architecture alongside eDNA. There are many parallels identified between the proteins we detected in the bands unique to the biofilm matrix compared to those identified by Dubois et al. in response to DOC^29^ and genes identified as upregulated in biofilm cultures by Poquet et al.^28^, including the cell surface proteins, amino acid metabolism and degradation, and fermentation, particularly butyrate. In *S. aureus*, positively charged cytoplasmic proteins^86^, eDNA-binding proteins and membrane-attached lipoproteins released into the extracellular environment by cell lysis make electrostatic interactions with the negatively charged cell surface proteins and eDNA to produce an electrostatic net, enhancing attachment and forming a stable biofilm matrix^13,82^. We identified the low molecular weight (LMW) cell wall protein SlpA as the most abundant protein within the biofilm matrix unique bands, which has been shown to enhance adhesion of vegetative *C. difficile* cells to host epithelial cells^87^ and potentially promote biofilm formation. Other proteins identified within the biofilm architecture were involved in amino acid fermentation, particularly Stickland fermentation including PrdA, which was also upregulated in *C. difficile* biofilms formed in response to DOC^29^. Other matrix proteins were involved in nitrogen and glutamate metabolism, including GluD, which facilitates resistance to H_2_O_2_ and found in the stool of CDI patients^88^ and Buk, involved in butyrate fermentation. Interestingly, we identified proteins involved in detoxification, most notably the rubrerythrin protein Rbr, involved in oxidative stress response, which complements data from Dubois et al*. *showing an upregulation of stress associated genes in *C. difficile* biofilms formed in the presence of DOC^29^. Other biofilm matrix proteins identified in the four unique bands include the chaperone DnaK, which is important in biofilm formation in *E. coli*^89^ and *C. difficile*^38^. This provides additional evidence of the presence of both cell wall and intracellular proteins within the *C. difficile* biofilm matrix.

There are a number of sources of eDNA and intracellular proteins within biofilms, including autolysis (*E. faecalis, S. aureus, P. aeruginosa, S. epidermidis*)^64^, phage induced lysis (*S. pneumoniae*, *Shewanella oneidensis*)^90,91^, active secretion^66^ and extracellular vesicles (OMVs), although the contribution of OMVs to biofilm formation is unclear^92^. Recent data suggests a role for the autolysin Cwp19 in biofilm formation in *C. difficile* in the presence of DOC^29^. Cwp19 has been shown to be involved in autolysis of *C. difficile* in stationary phase in BHIS media^93^. We observed Cwp19 and a phage protein (phiCD24-1) by LC–MS analysis of the matrix specific proteins, indicating that an autolysin and/or phage mediated cell lysis potentially contributes to the release of eDNA, in combination with lysis of sporulating cells driven by Spo0A. Other species such as *Cryptococcus neoformans* enhance biofilm formation by actively shedding their capsular polysaccharide glucuronoxylomannan^94^, therefore we could hypothesise that some of the cell surface proteins identified in the biofilm matrix maybe actively shed by *C. difficile*. We could also hypothesize that in a similar way to *S. aureus,* cytoplasmic proteins released during stationary phase may be recycled^86^ to form the architecture of the biofilm matrix in combination with eDNA and cell surface associated proteins, and that eDNA stabilizes the biofilm structure and contributes to antimicrobial resistance.

We show that eDNA is essential for the formation and structural integrity of *C. difficile* biofilms in all five *C. difficile* lineages, and that there is a positive correlation between biofilm biomass, eDNA and sporulation frequency. We hypothesize that the biofilm scaffold, comprised of eDNA, cell wall and intracellular proteins, surrounding vegetative cells and spores is most likely a result of cell lysis at stationary phase when *C. difficile* undergoes sporulation driven by the sporulation master regulator Spo0A, in combination with lysis of a subpopulation of cells via Cwp19 or phage mediated autolysis. Biofilms provide a potential reservoir of bacteria and spores to re-colonise an individual post-treatment, therefore we present a strategy using DNase to interfere with both biofilm initiation, maturation and recalcitrance for *C. difficile*, which reduces spore viability and promotes the efficacy of a current antibiotic therapy, such as vancomycin: which may prove valuable in the treatment of *C. difficile* infection or relapse.

## Materials and methods

### Growth of bacterial strains

*Clostridioides difficile* strains used in this study are summarized in Table 1^68,95,96^. Strains were cultured on either Brazier’s agar (Bioconnections, Leeds, South Yorkshire, UK) plus 4% egg-yolk, *C. difficile* supplement (Bioconnections) and 1% defibrinated horse blood or Brain heart infusion media (Oxoid) with 0.5% w/v yeast extract (Sigma) and 0.1% l-Cysteine (Sigma) (BHIS). Liquid cultures (primary cultures) were grown in BHIS broth containing BHI medium (Oxoid) plus yeast extract (0.5%) and 0.1% l-Cysteine in vented tissue culture (TC) flasks (25 cm^3^, Falcon), shaking at 65 rpm for 16 h, these were back diluted in pre-reduced media to OD_595nm_ 0.5 for dilution 1/10 into biofilm assays. All cultures were grown at 37 ºC in an anaerobic atmosphere (10% CO_2_, 10% H_2_, 80% N_2_) at 37 °C. When required 15 µg/mL thiamphenicol was added to retain the plasmid containing an inducible diguanylate cyclase (*dccA*). Expression of *dccA* was induced with either 25 ng/mL, 50 ng/mL, 100 ng/mL or 250 ng/mL anhydrotetracycline (ATc). This was added every 24 h to retain induction.

**Table 1:** List of strains and primers.

Strain	Description	Source
***E. coli strains***		
*E.coli* DH5α	General cloning	New England Biolabs
*E. coli* CA434	Conjugation donor	^97^
***C. difficile strains***		
630Δerm	Erythromycin-sensitive derivative of wild-type *C. difficile* 630	^98^
630Δerm_vector	Erythromycin-sensitive derivative of wild-type *C. difficile* 630 with empty plasmid pLFDempty	This study
630 (RT012)	Wild type ErmR, virulent and multidrug-resistant PCR ribotype	^51^
630_vector	Wild type ErmR, virulent and multidrug-resistant PCR ribotype containing pLFDempty	This study
R20291 (RT027)	Clinical isolate, virulent and multidrug-resistant PCR ribotype	^98^
CD305 (RT023)	Clinical isolate	^98^
M68 (RT017)	Clinical isolate	^98^
M120 (RT078)	Clinical isolate—non motile	^98^
CD630_0183::CT	Clostron mutant CD0183-549|550s	This study
CD630_2831::CT	Clostron mutant CD2831-1962|1963s	This study
CD630_3145::CT	Clostron mutant CD3145-482|483a	^58^
CD630_3392::CT	Clostron mutant CD3392-840|841s	This study
CD0183::CT (pCD0183)	Clostron mutant complemented with ptet inducible gene	This study
CD2831::CT (pCD2831)	Clostron mutant complemented with pcwp2 constitutively expressed Strep-tagged gene	This study
CD3145::CT (pCD3145)	Clostron mutant complemented with pcwp2 constitutively expressed Strep-tagged gene	^58^
CD3392::CT (pCD3392)	Clostron mutant complemented with pcwp2 constitutively expressed Strep-tagged gene	This study
630 pcwp2 *dccA*	*C. difficile* carying a pRFP144 derivative plasmid carrying Pcwp2-dccA for constitutive expression of DccA	^49^
630 ptet *dccA*	*C. difficile* carying a pRFP144 derivative plasmid carrying ptet -dccA for inducible expression of DccA	^49^

Plasmids	Description	Source
pCD0183	Retargeted pMTL007C-E2	This study
pCD2831	Retargeted pMTL007C-E2	This study
pCD3392	Retargeted pMTL007C-E2	This study
pRPF144	*E. coli-C. difficile* shuttle vector for protein expression. Constitutive Cwp2 promoter (Pcwp2) expressing gusA,TmR (5)	This study
pRPF185	*E. coli-C. difficile* shuttle vector for protein expression. Anhydrotetracycline inducible promoter (Ptet) expressing gusA, TmR	^99^
pLFDempty	pRPF185 containing ptet, with *gusA* removed	^99^
pHAS007	pRPF144 containing the *slpA* SecA2 secretion signal followed by a Strep II tag and an XhoI site for insertion of genes downstream	^58^
pHAS035 (p0183_comp)	pRPF185 containing the coding region of native *CD630_0183*	This study
pHAS013 (p2831_comp)	pHAS007 containing the coding region of *CD630_2831* from its signal cleavage site	This study
pHAS033 (p3145_comp)	pHAS007 containing the coding region of *CD630_3145* from its signal cleavage site	^58^
pHAS025 (p3392_comp)	pHAS007 containing the coding region of *CD630_3392* from its signal cleavage site	This study
pECC12	pRFP144 derivative carrying Pcwp2- *dccA* with a 3′ His-tag, for constitutive expression of DccA	^49^
pECC17	pRFP185 derivative carrying Ptet-dccA with a 3′ His-tag, for inducible expression of DccA	^49^

Primers	Sequence	Use
RAM-F	ACGCGTTATATTGATAAAAATAATAATAGTGGG	Identification of the clostron RAM cassette
RAM-R	ACGCGTGCGACTCATAGAATTATTTCCTCCCG	Identification of the clostron RAM cassette
CD0183-F	GGCGAAGGTTGGTTAGCTACTAG	Screening for CD630_0183 mutants
CD0183-R	GATGTTCTTGGTATATTCTTTCCTACTGC	Screening for CD630_0183 mutants
CD2831-F	CTTATACATTAAAGGTAATTAGTATTGAAGATAGC	Screening for CD630_2831 mutants
CD2831-R	CGTATAGTCTCCCCAACTTTTACATTTG	Screening for CD630_2831 mutants
CD3145-F	GCTAGAGAAAGTTAGCGCAATAATGC	Screening for CD630_3145 mutants
CD3145-R	CTATAGTATCTGCGAAAGTATTTGACGC	Screening for CD630_3145 mutants
CD3392-F	GAAATGCAAAACTGCAAAAAACCTCCAG	Screening for CD630_3392 mutants
CD3392-R	GATTGTCTTTGTAACCCATGTACGAG	Screening for CD630_3392 mutants
EBS Universal	CGAAATTAGAAACTTGCGTTCAGTAAAC	Screening for clostron mutants
CD0183-comp_F	GAGCTCGAAAATTTTAGGAGGTTTATCG	Complement construction
CD0183-comp_R	GGGATCCTTATAATATTCTTTTTGCTGTAACAAATCTTG	Complement construction
CD2831-comp_F	GGGGCTCGAGTCAGAATTAGGAGAGAATAGTCAGATTCAAAG	Complement construction
CD2831-comp_R	GGGGATCCCTAATTTGTATTTTTATTTCTTCTTAATACGATAAGTCCTAC	Complement construction
CD3145-comp-F	GGGGCTCGAGGATACTATAGAAGAAAGTACTAATGCAG	Complement construction
CD3145-comp-R	GGGGACTAGTTTATTTACGTCTTAAGTATTTATTTGTTAGATTTTTAATTA	Complement construction
CD3392-comp-F	GGGGCTCGAGGAAAGTAAGCAATACTGGACGGAAAG	Complement construction
CD3392-comp-R	GGGGGGATCCTTATGATTTCTTCATTTTACGGCGTTTATAAAG	Complement construction

### Gene inactivation in *C. difficile*

Isogenic mutants were constructed using either the ClosTron or CodA system (Table 1; Supplementary Fig. S4 online)^49,51,58,95,97–99^ in *C. difficile* strain 630Δ*erm*^95^ or 630, respectively. These mutants were used alongside the previously published C*D3145* Clostron mutant and complement^58^. For the Clostron mutant, the group II Ll.LtrB intron was retargeted to the gene of interest by SOE-PCR as previously described^100^, with oligonucleotides (listed in Table 1) designed using the Sigma TargetTron website (http://www.sigma-genosys.com/targetron/website). PCR products were cloned into *HindIII* and *BsrGI* sites of pMTL007C-E2 to create the plasmids pCD0183, pCD2831, and pCD3392 (Table 1). Retargeted pMTL007C-E2 plasmids were transformed into the *E. coli* conjugation donor strain CA434^97^ and transferred into *C. difficile* strain 630Δ*erm* by conjugation then screened for successful mutants as outlined previously^100,101^. DNA extracted from potential mutants (lincomycin resistant, thiamphenicol sensitive colonies), were screened by PCR and sequenced across the insertion/deletion site using gene specific primers or a gene specific primer with EBS universal primer, to verify insertion site. Mutants *CD3392* and *CD2831* were screened by Southern blot. Southern blot analyses were performed using AlkPhosDirect Labelling and detection kit (GE Healthcare) and detection reagents, in accordance with the manufacturer's guidelines and visualised using CDP star (GE Healthcare). Genomic DNA from wild type and potential mutants was digested with *BbaB1* for the *CD2831* screen (Southern band size for the mutant 3.8 Kb) or *BtgI* and *Pcil* for the *CD3392 *screen (Southern band size for the mutant 3.5 Kb). The probe was produced by PCR using RAM F and R primers (Table 1), from within the group II intron sequence. Mutations were complemented on a plasmid as outlined previously^100^ (Table 1).

### Biofilm formation

#### Attachment

This assay was performed in 24-well plates pre-equilibrated with 2 mL BHIS medium. The plates were incubated under anaerobic conditions for 6 h before inoculation at a 1/10 dilution with standardized primary cultures (overnight cultures in BHIS broth back diluted to OD_595nm_ 0.5) of *C. difficile* to optical density readings OD_595_ = 0.05 ± 0.01. Three control wells were left blank, without addition of bacterial cultures. Thiamphenicol (15 µg/mL) and anhydrotetracycline (25, 50, 100 or 250 ng/mL) were added as required. Plates were incubated for 16 h. The plates were then inverted to remove any cells that were not tightly attached to the abiotic surface, meaning the majority of the early stage biofilm is removed, leaving only the attached cells. Each well was then washed twice with 1 mL PBS. The liquid was removed by inversion and tapping between each step. The plates were allowed to air-dry in the anaerobe cabinet for 10 min before the attached cells were stained with 800 µL of 0.1% crystal violet for 30 min. Plates were washed four times with 1 mL PBS, inverting plates between each step, and the crystal violet was solubilized with 1 mL methanol. Attachment was determined using OD_595_ readings, with a subtraction of the mean of three blanks present on each individual plate. Assays were performed with a minimum of six independent replicates. The data was analysed in Excel and GraphPad prism 7.0. Statistical analysis was performed (see below).

#### Early biofilm formation

Biofilms were produced by inoculating overnight cultures of *C. difficile* into 24-well plates containing 2 mL pre-equilibrated BHIS (+ 0.1% l-Cysteine) per well at OD_595_ = 0.05 ± 0.01, with the exception of three control wells, left un-inoculated. All strains were compared to *C. difficile* control strain 630, carrying a plasmid without *dccA*. These cultures were incubated statically for 24 h, after which supernatants were carefully removed with a pipette to not disturb the early biofilm attached to the plate. These biofilms were washed twice with 800 µL 1X PBS, before the addition of 800 µL of 0.1% crystal violet. The plates were washed four times with 1X PBS and the crystal violet was solubilized with 1 mL methanol. The OD_595_ readings were obtained and the mean of three blank wells present on each individual plate was subtracted. As required, cultures were supplemented with thiamphenicol and/or anhydrotetracycline (ATc). Biofilm biomass was determined using OD_595_ readings of the methanol solubilized crystal violet. A minimum of six independent replicates were performed. The data was analysed in Excel and GraphPad prism 7.0. Statistical analysis was performed (see below).

#### Late biofilm formation

Biofilms were produced as outlined above but were incubated statically for 72 h to enable maturation. As required, media were supplemented with thiamphenicol and/or ATc. Biofilm biomass was determined using OD_595_ readings of the methanol solubilized crystal violet. The mean OD_595_ reading of three blank wells present on each individual plate was subtracted from each sample well. A minimum of six independent replicates were performed. The data was analysed in Excel and GraphPad Prism 7.0. Statistical analysis was performed (see below).

#### Statistical analysis of biofilm biomass

Biofilm formation measured by crystal violet assays outlined above was assessed using a Students t test or Linear Regression Analysis in Stata IC 15^23^. For linear regression, the data was transformed using log_10_ to approximate a normal distribution before a Linear Regression and partial F tests were performed to answer the following questions:

- Is there strong evidence that the concentration of eDNA or total DNA contained within a biofilm matrix varies between strains? Linear regression was used to determine if there is strong evidence that there are differences in eDNA concentration in biofilm biomass linked to strain, where *p* < 0.05 indicates strong evidence a biofilm contains less eDNA or total DNA within the biofilm matrix compared to the control strain (630). The coefficient of variance (COV) (used throughout) determines whether this difference is higher (positive number) or lower (negative number) than the reference (630) (COV = 0) (Table 2).
- Is there strong evidence that cell surface proteins play a role in biofilm formation, measured by biofilm biomass. Linear regression was used to determine if there is strong evidence that there are differences in biofilm biomass taking individual strains into account. A *p* < 0.05 indicates strong evidence that deletion of cell surface genes or overexpression of cell surface genes, affect biofilm biomass, relative to their specific wild-type strains (630∆*erm* or 630). (Table 2).
- Is there strong evidence that the addition of c-di-GMP influences biofilm biomass in attachment, early biofilm or late biofilm formation, compared to the un-induced control strain (630 *ptet-dccA* NI). A partial F test was performed to determine if c-di-GMP had an effect on biofilm biomass (*p* < 0.05). Linear regression was used to determine if there is strong evidence that differences in biofilm biomass are linked to induction of *dccA*, where a *p* < 0.05 indicates strong evidence that c-di-GMP levels, driven by induction of *dccA* affect biofilm biomass compared to the control (Table 2).
- Is there strong evidence that the strain of *C. difficile* influences biofilm biomass? A partial F test and linear regression were performed to determine if there were strain specific differences in biofilm formation using representatives of the five *C. difficile* clades. *p* < 0.05 indicates strain specific differences (Table 2).
- Is there strong evidence that there are strain specific differences in vegetative cells and spores between different *C. difficile* lineages compared to strain 630. Is there strong evidence that percentage sporulation within a biofilm differs between ribotypes of the five main lineages. Where a *p* < 0.05 indicates strong evidence of strain specific differences in viable cell counts or sporulation frequency.

**Table 2:** Statistical analysis.

DNA concentration in the biofilm matrix		Regression analysis		Partial F test
Conditions	Strain	COV	*p* value	p value
Extracellular DNA:	630	0	Reference	
	R20291	**− 0.371**	**0.038**	**0.0382**
Total DNA:	630	0	Reference	
	R20291	−0.1	0.53	**0.5302**

Cell surface proteins	Regression analysis		Partial F test	
Strain	COV	*p* value	p value	
630∆erm	0	Reference	**0.0082**	
CD*2831*::CT	− 0.401	**0.019**		
CD*2831*::CT::*P*_*tet*_*2831*	0.187	0.265		
630	0	Reference	**0.0143**	
630 ∆*PEPP-1*	− 0.09	0.623		
630 ∆*PEPP-1*::P_*tet*_*2831*	0.427	**0.011**		
630 ∆*PEPP-1*::P_*tet*_*3246*	0.434	**0.029**		
630∆erm	0	Reference	**0.0004**	
*CD0183*::CT	− 0.979	**0.000**		
*CD0183*::CT::P_*tet*_*0183*	− 0.273	0.184		
*CD3145*::CT	− 0.453	**0.029**		
*CD3145*::CT::*p3145*	− 0.511	**0.014**		
*CD3392*::CT	− 0.64	**0.002**		
*CD3392*::CT::*p3392*	− 0.277	0.178		

c-di-GMP	Strain	Regression analysis		Partial F test
		COV	*p* value	p value
Attachment	630 P_*tet*_-*dccA* NI	0	Reference	0.1957
	630 empty	0.065	0.753	
	630 P_tet_-*dccA*25	0.163	0.354	
	630 P_tet_-*dccA*100	− 0.319	0.264	
	630 vector	0	Reference	0.3339
	630 vector + ATc	0.185	0.334	
Early biofilm	630 P_*tet*_*-dccA* NI	0	Reference	**0.0059**
	630 empty	0.438	0.108	
	630 P_*tet*_-*dccA*25	**0.910**	**0.001**	
	630 P_*tet*-_*dccA*100	**0.799**	**0.004**	
	630 vector	0	Reference	0.4876
	630 vector + ATc	0.153	0.488	
Mature biofilm	630 P_*tet*_-*dccA* NI	0	Reference	0.3946
	630 empty	0.314	0.538	
	630 P_*tet*_-*dccA*25	− 0.538	0.295	
	630 P_*tet*_-*dccA*100	− 0.243	0.634	
	630 vector	0	Reference	0.1047
	630 vector + ATc	− 0.340	**0.105**	

**Biofilm disruption**	Strain	Regression analysis		Partial F test
		COV	*p* value	p value
Vegetative cells	Control	0	Reference	**0.0007**
	Control + Vancomycin	− 6.243	**0.013**	
	DNase + Vancomycin	− 10.805	**0.000**	
	Proteinase K + vancomycin	− 0.309	0.916	
Spores	Control	0	Reference	0.3032
	Control + Vancomycin	− 0.09	0.776	
	DNase + Vancomycin	− 0.312	0.330	
	Proteinase K + vancomycin	− 0.585	0.074	
Vegetative cells	Control + Vancomycin	0	Reference	**0.0020**
	DNase + Vancomycin	− 1.662	**0.021**	
	Proteinase K + vancomycin	1.927	**0.007**	
Spores	Control + Vancomycin	0	Reference	**0.0281**
	DNase + Vancomycin	− 0.222	0.195	
	Proteinase K + vancomycin	− 0.494	**0.009**	
Vegetative cells	Control	0	Reference	0.9849
	DNase	0.010	**0.989**	
	Proteinase K	− 0.086	**0.897**	
Spores	Control	0	Reference	0.0142
	DNase	− 0.568	**0.050**	
	Proteinase K	0.437	**0.109**	

Five main *C. difficile* lineages biofilm biomass	Regression analysis		Partial F test	
Strain	COV	*p* value	p value	
630 (RT012)	0	Reference	**0.0072**	
R20291 (RT027)	− 0.449	**0.050**		
CD305 (RT023)	− 0.671	**0.003**		
M68 (RT017)	− 0.026	0.907		
M120 (RT078)	0.092	0.746		

Total CFU counts in matrix compared to the supernatants		Regression analysis		Partial F test
Cell type	Strain	COV	*p* value	p value
Vegetative cells-strain 630	Matrix	0	Reference	0.2423
	Supernatant	0.29	0.242	
Spores-strain 630	Matrix	0	Reference	**0.0000**
	Supernatant	**− 4.29**	**0.000**	
Vegetative cells-strain R20291	Matrix	0	Reference	**0.0000**
	Supernatant	**2.91**	**0.000**	
Spores-strain R20291	Matrix	0	Reference	**0.0023**
	Supernatant	**− 1.72**	**0.002**	
Vegetative cells-strain M210	Matrix	0	Reference	0.2174
	Supernatant	− 0.92	0.217	
Spores-strain M120	Matrix	0	Reference	**0.0001**
	Supernatant	**− 1.58**	**0.000**	
Vegetative cells-strain M68	Matrix	0	Reference	0.1931
	Supernatant	0.20	0.193	
Spores-strain M68	Matrix	0	Reference	**0.0000**
	Supernatant	**− 1.55**	**0.000**	
Vegetative cells-strain CD305	Matrix	0	Reference	**0.0107**
	Supernatant	**− 1.08**	**0.011**	
Spores-strain CD305	Matrix	0	Reference	0.6668
	Supernatant	− 0.16	0.667	

DNA concentration in the biofilm matrix		Regression analysis		Partial F test
Conditions	Strain	COV	*p* value	p value
24 well plates	630 (RT012)	0	Reference	**0.0282**
	R20291 (RT027)	− 0.371	**0.050**	
	CD305 (RT023)	− 0.168	0.303	
	M68 (RT017)	− 0.735	**0.004**	
	M120 (RT078)	− 0.345	0.066	
TC flasks	630 (RT012)	0	Reference	**0.0045**
	R20291 (RT027)	− 0.540	**0.039**	
	CD305 (RT023)	− 0.163	0.459	
	M68 (RT017)	− 1.194	**0.001**	
	M120 (RT078)	− 0.025	0.897	

Percentage sporulation in the biofilm matrix		Regression analysis		Partial F test
Conditions	Strain	COV	*p* value	p value
Biofilm matrix	630 (RT012)	0	Reference	
	R20291 (RT027)	**− 0.0914**	**0.000**	
	CD305 (RT023)	0.171	0.584	
	M68 (RT017)	**− 0.818**	**0.008**	
	M120 (RT078)	0.351	0.193	**0.0003**

Showing the Students T test *p* values, the Partial F test *p* value for the groups analysed by linear regression and linear regression Coefficient of Variance and *p* values.

### Structural components of the biofilm matrix.

#### SDS-PAGE and Western blot

Primary overnight cultures of 630Δ*erm*, *CD3392*::CT and the complement *CD3392*:: (p*CD3392*) were diluted to OD_595nm_ 0.5, then inoculated 1/10 to give a final OD_595_ 0.05 ± 0.01 into TC flasks (Falcon), and static biofilm cultures were grown for three days (late biofilms) under anaerobic conditions. Carefully without disturbing the biofilm matrix, 1 mL of the supernatant (planktonic fraction) was removed from the static biofilm culture and transferred to a 1.5 mL microcentrifuge tube, then samples were filter sterilised (0.2 µM filter) to remove viable cells and spores. The biofilm matrix was detached from the base of the TC flask by gentle agitation^23^ and removed intact using a 1 mL pipette tip before being transferred to a fresh 1.5 mL microcentrifuge tube (as outlined in Fig. 6a). The intact biofilm matrix was disrupted by digestion with DNase for 15 min at room temperature (R/T) with 10 µg/mL DNase I. The planktonic and matrix fractions (obtained using DNase I digestion of the biofilm matrix) were visualised on an SDS-PAGE gel (data not shown). In parallel CFU counts were obtained from the planktonic and matrix fractions at early biofilm (24 h—planktonic fraction (2.48 × 10^8^ vegetative cells, 1.91 × 10^4^ spores) and matrix fraction (2.33 × 10^5^ vegetative cells, 1.17 × 10^6^ spores)) and late biofilm [3 days—planktonic fraction (3.94 × 10^5^ vegetative cells, 2.35 × 10^3^ spores) and matrix fraction (2.57 × 10^5^ vegetative cells, 1.78 × 10^5^ spores)]. The iBlot 2 Dry Blotting System (Thermofisher) was used for transfer of samples from the SDS-PAGE gel to a nitrocellulose membrane (Thermofisher). Transfer stacks were assembled and loaded onto the iBlot according to manufacturer’s instructions and transferred for 7 min at 20 V. Following transfer, membranes were washed for 5 min in 1 × PBS + 0.1% Tween20 then incubated with blocking buffer (1 × PBS + 0.1% Tween20 and 5% skimmed milk) for 1 h, at room temperature on a rocking platform (Stuart). Primary antibody (anti-CD3392 antibody) was added at 1:250 dilution into fresh blocking buffer and incubated for 1 h. Membranes were washed 3 × 5 min with 1 × PBS + 0.1% Tween20 before incubation with the fluorescently label goat anti mouse secondary antibody 1/5000 at the appropriate dilution, in the dark. The membrane was washed 3 × in 1 × PBS + 0.1% Tween20 before visualisation on a LI-COR. Images were scanned at 680 nm and labelled in Photoshop elements to retain image resolution.

#### Protein sample preparation and analysis

Primary overnight cultures of *C. difficile* strains were diluted to OD_595nm_ 0.5, then inoculated 1/10 to give a final OD_595_ 0.05 ± 0.01 into TC flasks (Falcon), and static biofilm cultures were grown for three days under anaerobic conditions. 1 mL of the supernatant (planktonic fraction) was transferred to a 1.5 mL microcentrifuge tube for processing, then the biofilm matrix was detached from the base of the TC flask by gentle agitation^23^ and removed intact using a 1 mL pipette tip before being transferred to a fresh 1.5 mL microcentrifuge tube for processing (Fig. 6a). The biofilm matrix was disrupted by digestion with DNase for 15 min at room temperature (R/T) with 10 µg/mL DNase I. The planktonic and matrix samples were filter sterilised (0.2 µM filter) to remove viable cells and spores. A duplicate late biofilm (72 h) was processed for protein quantification, herein, the supernatant and matrix were separated as outlined above, however, for protein quantification the matrix samples were vortexed for 2 min to disrupt the biofilm (without the addition of DNase I). The total protein concentration in the planktonic fraction (supernatant) (765 µg/mL) and matrix (793 µg/mL) fractions were determined using a BCA assay (Pierce BCA assay kit) in accordance with the manufacturer’s instructions. The planktonic and matrix fractions (obtained using DNase I digestion of the biofilm matrix) were visualised on an SDS-PAGE gel, with a DNase I only control lane (Fig. 6). The four bands unique to the matrix fraction were excised and sent for LC–MS/MS analysis (King’s College London, CEMS Proteomics facility) (Fig. 6). In-gel reduction, alkylation and digestion with trypsin were performed on the excised gel bands prior to subsequent analysis by mass spectrometry. Cysteine residues were reduced with dithiothreitol and derivatized by treatment with iodoacetamide to form stable carbamidomethyl derivatives. Trypsin digestion was carried out overnight at R/T after initial incubation at 37 °C for 2 h. Peptides were extracted from the gel pieces by a series of acetonitrile and aqueous washes. The extract was pooled with the initial supernatant and lyophilised. The sample was then re-suspended in 10 μL of resuspension buffer (2% ACN in 0.05% FA) and analysed by LC–MS/MS. Chromatographic separation was performed using a U3000 UHPLC NanoLC system (ThermoFisherScientific, UK). Peptides were resolved by reversed phase chromatography on a 75 μM C18 column (15 cm length) using a three-step linear gradient of 80% acetonitrile in 0.1% formic acid. The gradient was delivered to elute the peptides at a flow rate of 250 nL/min over 60 min. The eluate was ionised by electrospray ionisation using an Orbitrap Fusion Lumos (ThermoFisherScientific, UK) operating under Xcalibur v4.1.5. The instrument was programmed to acquire in automated data-dependent switching mode, selecting precursor ions based on their intensity for sequencing by collision-induced fragmentation using a TopN CID method. The MS/MS analyses were conducted using collision energy profiles that were chosen based on the mass-to-charge ratio (*m/z*) and the charge state of the peptide. Raw mass spectrometry data were processed into peak list files using Proteome Discoverer (ThermoScientific; v2.2). The raw data file was processed and searched using the Mascot search algorithm (v2.6.0; www.matrixscience.com) and the Sequest search algorithm (Eng et al.; PMID 24226387) against the current All Taxonomy and *C. difficile* databases from Uniprot, at a stringency threshold of 5% false discovery rate (FDR) for protein and peptide and a minimum of one peptide per protein. The Database generated file was uploaded into Scaffold 4 (v4.8.7) software (www.proteomesoftware.com) for analysis. This was also repeated for Bovine DNase I. The stringency threshold parameters were therefore set to 95% protein, minimum 1 peptide and 95% peptide. The data from each band was then aligned with a protein classification (Riley classification) and processed in Excel (Supplemenatry Table S1). Pie charts were produced to show the percentage representation of proteins in the matrix.

#### DNA quantification

Primary overnight cultures of *C. difficile* strains 630, R20291, M120, M68 and CD305 were back diluted in pre-reduced BHIS + Cysteine to OD_595_ 0.5 and used to inoculate 1/10 into two matched vented tissue culture flasks (Falcon) containing 10 mL fresh BHIS + cysteine, or duplicate 24-well plates (Corning) with each well containing 2 mL fresh BHIS + cysteine at an inoculum density of OD_595_ = 0.05 ± 0.01. These cultures were incubated statically for three days. Biofilms were detached from the surface of tissue culture flask by gentle agitation (Fig. 6a), after which the intact biofilm was removed with a pipette within a volume of 1 mL and transferred to a 2 mL screw cap tube. For the 24-well plates, 1.5 mL of excess liquid was removed, then the biofilm was detached from the bottom of the well using a pipette and was re-suspended in 500 μL BHIS broth. Biofilms were vortexed to disrupt the biofilm matrix. Matched biofilms were processed in parallel to (1) extract matrix eDNA by removal of vegetative cells and spores by passing the disrupted biofilm through a 0.22 µM filter or (2) extraction of total DNA (matrix eDNA and intracellular DNA), disrupted biofilm matrix samples were transferred to a Lysis Tube B (MP Biomedical) before ribolysing at 6.0 m/s for 45 s using a FastPrep-24 classic (MP Biomedicals). Both Matrix eDNA and total DNA samples were then processed and extracted using the DNeasy Blood and Tissue Kit (Qiagen), following the manufacturers guidelines for the Purification of Total DNA from Animal Blood or Cells. In short, 500 µL of the sample from tissue culture flasks or 200 µL from 24-well plates were processed with the AL buffer in accordance with the manufacturer’s guidelines. Samples were eluted in 100 µL of elution buffer. DNA concentrations were then measured by spectrophotometry using a DS-11 FX+ (DeNovix) according to manufacturer’s instructions, and concentrations were normalized to per biofilm (mg/biofilm).

### Microscopy

#### Confocal microscopy

Thermanox plastic coated 13 mm coverslips (Fisher) were added to the 24-well plates containing BHIS media (as appropriate with ± thiamphenicol, ± anhydrotetracycline) prior to inoculation with *C. difficile* at OD_595_ 0.05 ± 0.01. After 24 or 72 h incubation, the media was carefully removed with a pipette and the coverslips were washed once in the wells with 1X PBS, before the addition of: 200 µL of Acridine Orange diluted to 1X in PBS (PRO-LAB diagnostics), 200 µL FilmTracer SYPRO Ruby (supplied as a 1X solution) (Thermo Fisher), or a drop containing 37.5 ng of 4′,6-diamidine-2′-phenylindole dihydrochloride (DAPI) Vectashield mounting media (Vector laboratories), or 200 µL of FilmTracer LIVE/DEAD biofilm viability kit, containing 4.45 nM Syto9 and 13 nM Propidium iodide (Life Technologies) onto the coverslips inside the wells and incubated for 30 min. Excess stain was removed and washed twice with 1X PBS. The stained thermanox coverslips were mounted face up onto glass slides, with 10 µL 30% glycerol under a glass coverslip (22 mm × 22 mm), sealed with clear nail varnish. Slides were visualised under oil immersion (40× and 100× objectives) using a laser confocal microscope (LSM510 Zeiss microscope). The excitation/emission used for these dyes were 543 nm/> 560 nm for Acridine orange, 358 nm/> 461 nm DAPI and FilmTracer LIVE/DEAD stain used 488 nm/> 560 nm (SYTO9) and 543 nm/> 650 nm (Propidium Iodide). Acridine orange was scanned at both the green (488 nm/> 560 nm) and red (543 nm/> 650 nm), as Acridine orange stains double stranded DNA green and ssDNA/RNA red. Z-stacks were performed to measure the biofilm depth. The assays were performed with a minimum of quadruplicate independent replicates, imaging a minimum of three fields of view per slide and images were analysed using Zeiss LSM image browser and Volocity imaging software.

#### Scanning electron microscopy

Triplicate independent 2 mL liquid cultures of 630 (in BHIS + cysteine) were inoculated into pre-equilibrated low evaporation lid 24-well plates (Nunc) containing Thermanox coverslips (Fisher), including two blank media controls. The plates were sealed with Nescofilm and incubated statically at 37 °C as outlined above. Coverslips were processed at 16 h, 24 h and 72 h for attachment, early biofilm formation and late biofilm formation, respectively. Coverslips were washed in 1X PBS before fixation. Alternatively, late biofilms were detached from the bottom of a TC flask, the biofilm was carefully extracted using a pipette (Fig. 6a), then added directly into 1 mL fixative before being transferred to a coverslip and stub for SEM imaging. The fixative was applied to coverslips or late biofilms, 1 mL fixative (2.5% Paraformaldehyde/2.5% Glutaraldehyde/0.1 M Sodium cacodylate pH 7.4) was added to each sample, including the media blank controls. The samples were washed in 0.1 M Na cacodylate, post-fixed in 1% aqueous/0.1 M Na cacodylate, and stored in MilliQ water overnight at 4 °C. These were then air dried and mounted onto an aluminium stub prior to sputter coating with gold. Samples were imaged using a JSM35 Scanning Electron Microscope. A minimum of three fields of view were imaged per slide at 1× K, 3.5× K, 5× K, 7× K and 10× K magnification for the biofilms grown on coverslips in 24-well plates. For the biofilms grown in TC flasks the samples were images at 150×, 1× K and 3× K and 10× K on a Scanning Electron Microscope.

### Inhibition and degradation of biofilms

#### Degradation of biofilms

Liquid cultures of *C. difficile* strains 630∆*erm*, 630, R20291, CD305, M120 and M68 were incubated in TC flasks under anaerobic conditions for 72 h as outlined above, then were removed from the anaerobe hood. The biofilms were detached from the bottom of the flask by gentle agitation. These intact biofilms were treated with 1 mg/mL and 100 µg/mL Recombinant DNase (Sigma) (10 mg stock dilutions were made in 0.15 M NaCl_2_), 1 mg/mL and 100 µg/mL RNase A (Sigma) (dilutions were made in nuclease free dH_2_0) or 1 mg/mL Proteinase K (Qiagen) (stock 20 mg/mL). Samples were incubated at R/T for 15 min. Before and after addition of the DNase, RNase or Proteinase K, images were taken using a Canon 600D SLR (50 mm prime lens), mounted on a Kaiser RS2 copy stand with lighting unit. Late biofilms produced on Thermanox coverslips as outlined above were incubated for 15 min with DNase (1, 10, 100 µg/mL and 1 mg/mL). The slides were processed for confocal microscopy using FilmTracer LIVE/DEAD biofilm viability kit as outlined above.

#### Inhibition of biofilm formation

Cultures of *C. difficile* strain 630∆*erm* were inoculated at OD_595_ 0.05 ± 0.01 as outlined above into TC flasks (Falcon) containing BHIS media with recombinant DNase (500 µg/mL or 1 mg/mL final concentration) as outlined above. Images were taken of the 72-h cultures flasks as outlined above.

#### The effect of vancomycin on CFU counts in disrupted and intact biofilms

The effect of vancomycin treatment on both vegetative cells and spores within an intact and degraded biofilm was undertaken in 3-day old biofilms. The biofilms were detached from the bottom of the flask by gentle agitation. These intact biofilms were treated with either 1 mg/mL recombinant DNase (Sigma), 1 mg/mL Proteinase K (PK) (Qiagen), 12.5 mg/mL Vancomycin, or a combination of these, compared to an untreated intact biofilm. Samples were incubated for 24 h at 37 °C in an anaerobic atmosphere. Samples were then pelleted by centrifugation at 4500×*g* for 10 min. Pellets were washed twice in 10 mL sterile PBS, then pelleted and re-suspended in 1 mL of 1× PBS and vortexed to break down any remaining intact biofilm. Total counts were obtained from 1 mL of each disrupted biofilm culture, serially diluted in 1× PBS and plated in triplicate onto BHIS plates supplemented with 0.1% taurocholate. Spores were isolated from total cells by heat inactivating 1 mL of each sample (65 °C for 25 min), killing the vegetative cells, and were plated in triplicate onto BHIS agar plus 0.1% sodium taurocholate. The data was analysed in Excel and GraphPad Prism 7.0. Regression analysis was performed to identify significant differences in total cell counts and spore counts caused by exposure to vancomycin in disrupted versus intact biofilms (see below).

#### Statistical analysis of the effect of vancomycin on the CFU counts

The CFU data were transformed using log_10_ to approximate a normal distribution, after which a linear regression analysis was performed to determine whether there was strong evidence that disruption of biofilms increased the vancomycin susceptibility of the vegetative cells and spores encased within compared to the intact biofilm. Analysis was undertaken to answer if there: were differences in viability of the vegetative cells (1) or spores (2) in the presence of vancomycin in intact and DNase or PK disrupted biofilms compared to the untreated biofilm control? These analyses were repeated to see if DNase and PK treatment of the biofilms made the (3) vegetative cells or (4) spores more susceptible to vancomycin than the non-disrupted vancomycin treated control. For all the analyses *p* < 0.05 indicates a significant difference and the coefficient (C) determines whether this difference is higher (positive number) or lower (negative number) than the reference (indicated in Table 2) (C = 0).

## Supplementary Information

Supplementary Figures.

Supplementary Table 3.
