# Supplementary material for: Extracellular DNA, cell surface proteins and c-di-GMP promote biofilm formation in Clostridioides difficile
Source: Sci Rep. 2021 Feb 5;11:3244. doi: 10.1038/s41598-020-78437-5 (PMC7865049; doi:10.1038/s41598-020-78437-5)
Supplement: Supplementary file 1 — Supplementary Figures. [file 41598_2020_78437_MOESM1_ESM.docx]

**Extracellular DNA, cell surface proteins and c-di-GMP promote biofilm formation in *Clostridioides difficile***

Lisa F. Dawson^1#^, Johann Peltier^1*^, Catherine L. Hall^1^, Mark A. Harrison^1^, Maria Derakhshan^1^, Helen A. Shaw^1*^, Neil F. Fairweather^2^ and Brendan W. Wren^1^


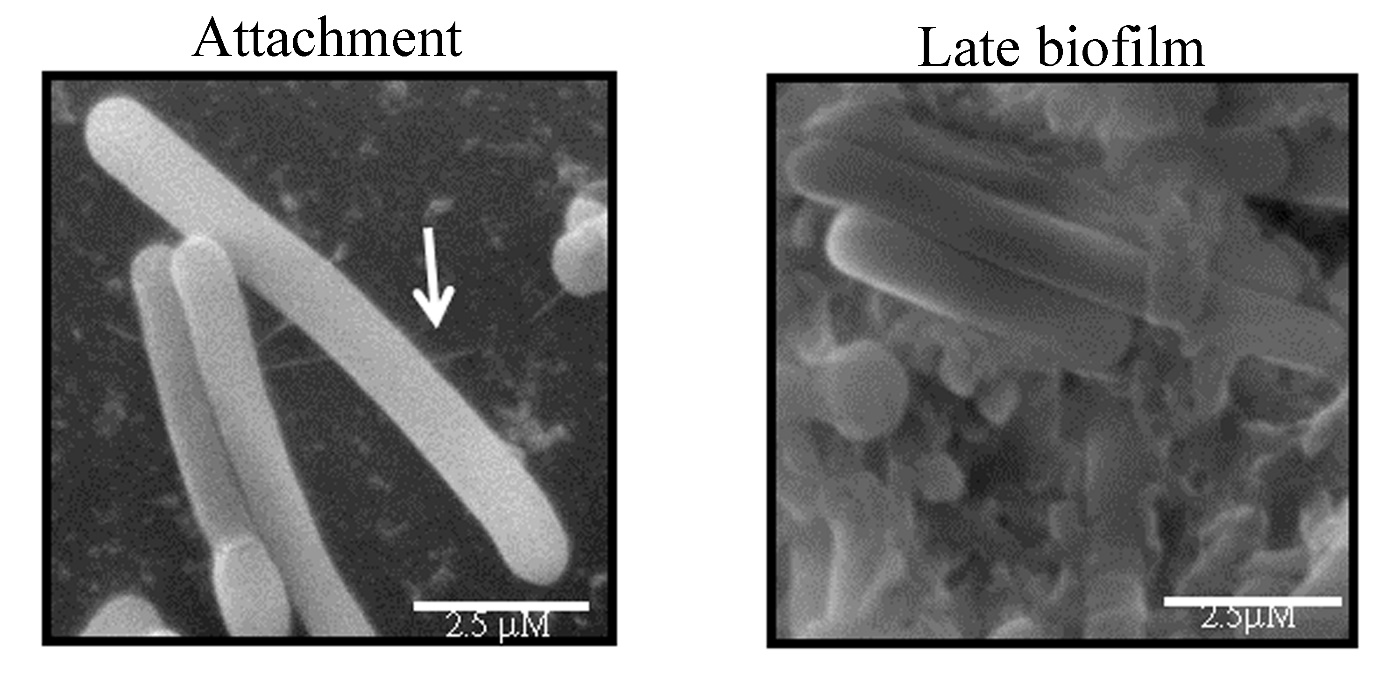


**Supplementary Figure 1: Magnified SEM visualisation of biofilm formation of *C. difficile* strain 630.** SEM magnification of attachment (16 hours) and late biofilms (72 hours) within the white box from Fig. 1. Scale bar = 2.5 µm.


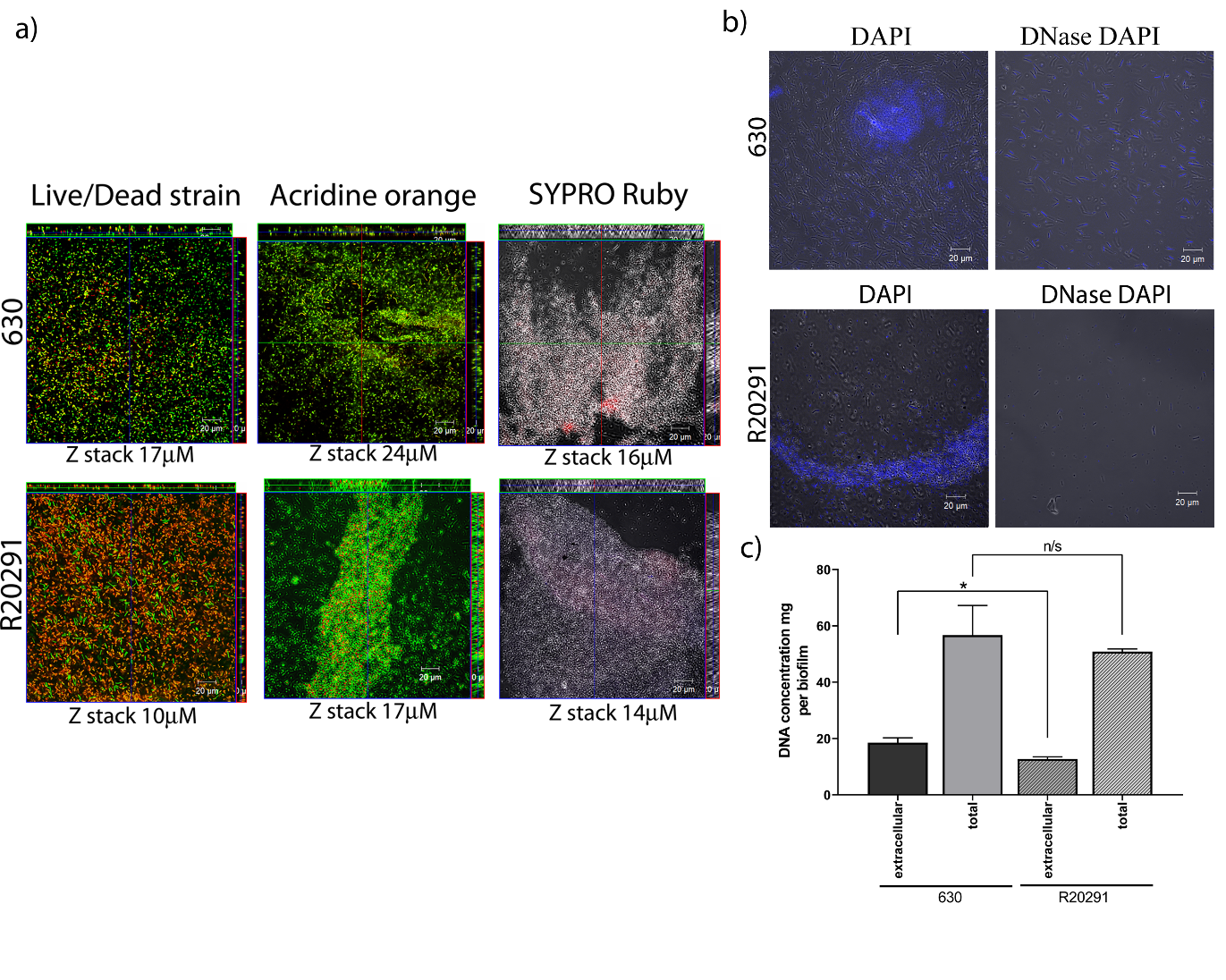


**Supplementary Figure 2: Confocal fluorescence microscopy of biofilm components and quantification of eDNA in a *C. difficile* biofilm***.* a) Biofilms were grown on Thermanox coverslips in 24-well plates, for strains 630 and R20291 and stained with either FilmTracer LIVE/DEAD biofilm viability stain, acridine orange (nucleic acid) or FilmTracer SYPRO Ruby biofilm matrix stain (protein). Acridine orange stains double stranded DNA green and single stranded DNA/RNA red. The size of the Z-stacks in μm is indicated below the confocal images. b) Confocal fluorescence microscopy of biofilms formed on Thermanox coverslips, for strains 630 (RT012) and R20291 (RT027) were stained with 4',6-diamidino-2-phenylindole (DAPI). Duplicate coverslips were incubated for 15 mins with 100 μg/ml DNase, to disrupt the eDNA within the biofilm and were imaged by confocal microscopy. c) The concentration of eDNA (filtered to remove cells) and total DNA (ribolysed to disrupt cells) was determined from the biofilm matrix. Error bars are SD. Statistical analysis was performed using Linear regression analysis to compare the overall DNA concentrations from biofilms formed in 24-well plates * p<0.05.


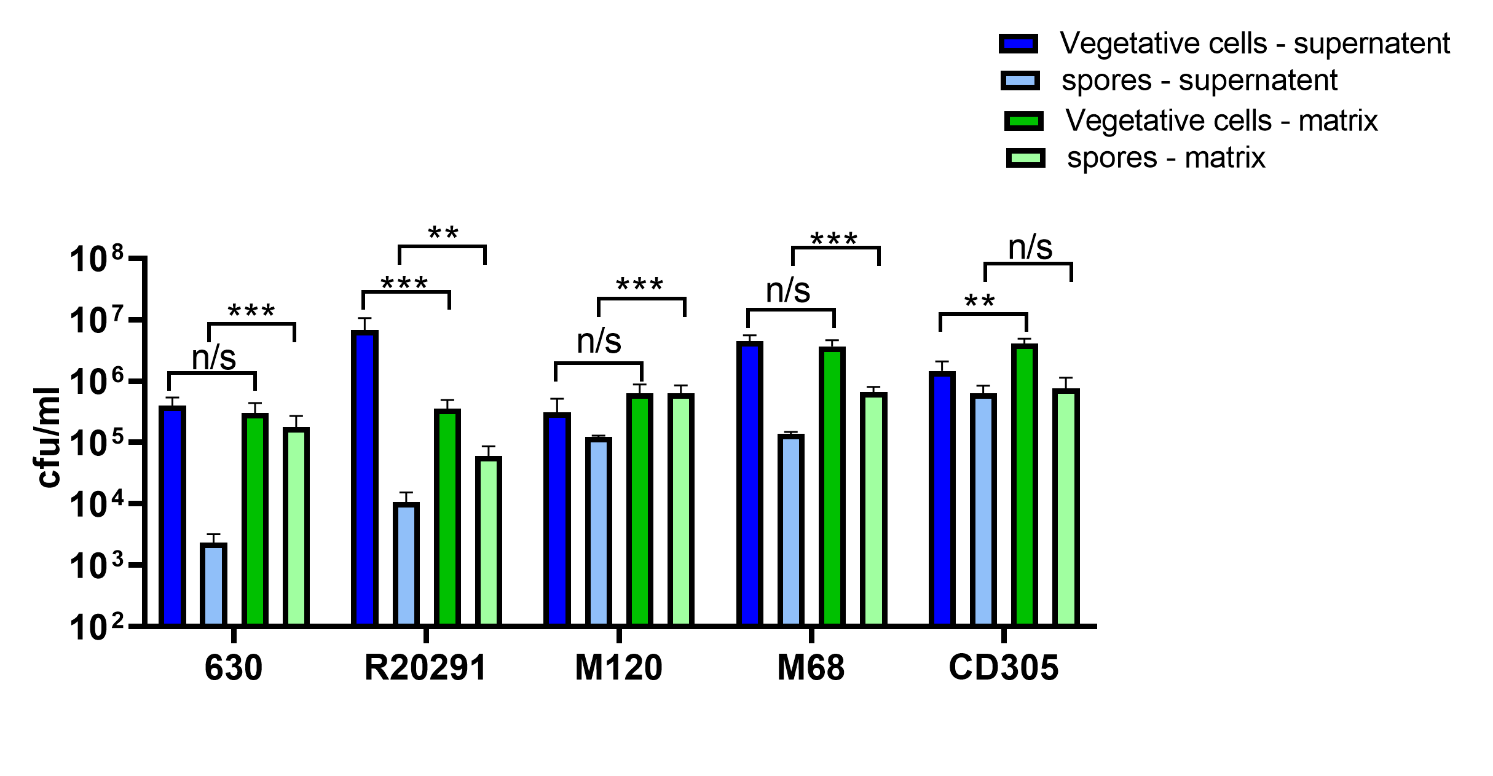


**Supplementary Figure 3:** **CFUs from biofilm matrix and planktonic phase following DNase treatment.**

Strains from all five lineages of *C. difficile* 630 (RT012), R20291 (RT027), M120 (RT078), M68 (RT017) and CD305 (RT023) strains were grown statically in tissue culture flasks for three days to allow biofilm development. A 1 ml aliquot of the planktonic phase (supernatant) was removed for CFU analysis, after which the biofilm was gently detached from the bottom of the TC flask and collected using a pipette in 1 ml volume. The biofilm was then disrupted with 100 µg/ml DNase before CFU counts were determined. The relative proportion of vegetative cells and spores were determined for the biofilm matrix compared to the supernatant. Error bars are SD. Significant differences between the vegetative cells or spores within the supernatant compared to the biofilm matrix were calculated using Linear regression analysis * p<0.05, ** p<0.01, *** p<0.001


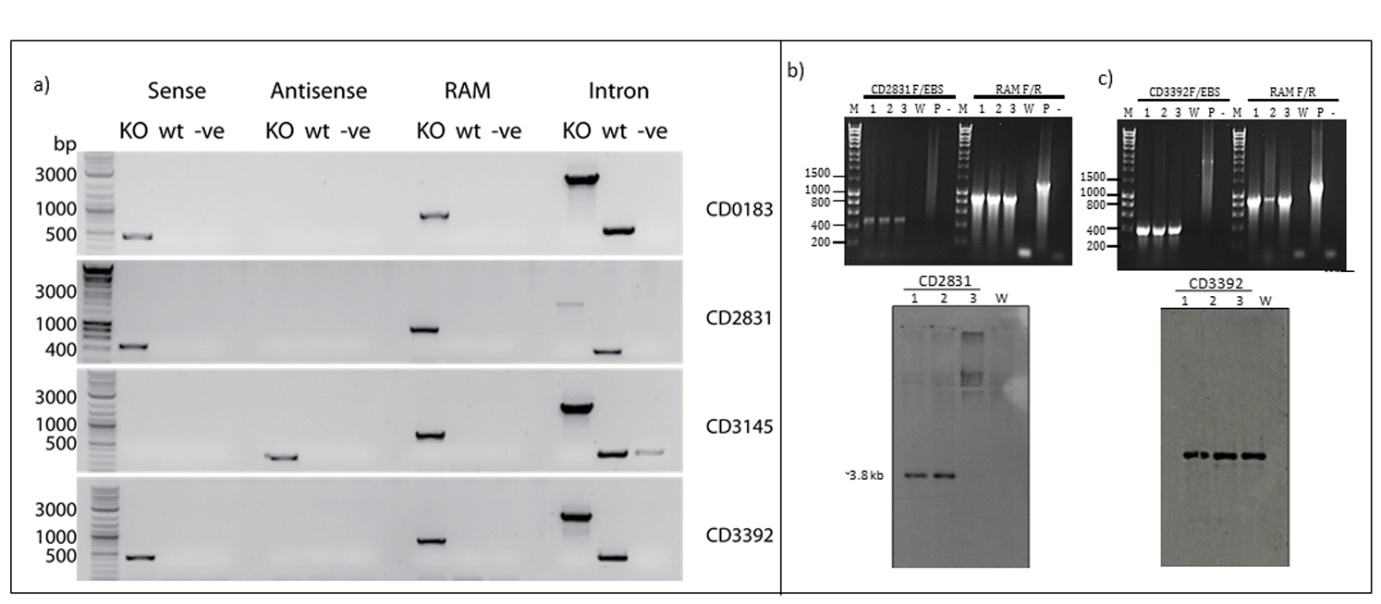


**Supplementary Figure 4:** **PCR screen and Southern blot analysis of clostron mutants**

a) PCR screen of the mutant strain compared to parental strain 630Δ*erm,* to detect the insertion of a Clostron carrying a RAM cassette for CD0183, CD2831, CD3145 and CD3391. PCR was performed using primers specific for the RAM and intron as well as to determine sense and antisense orientation of the Clostron insertion. b) A PCR screen and Southern blot to screen potential *CD2831* mutants using a probe specific to the inserted Ll.ltrB intron. *Btg*I and *Pci*I digests were performed on DNA from three clones each of potential *CD3392* mutants, and the parent strain 630Δ*erm*. c) A PCR screen and corresponding Southern blot to screen potential *CD3392* mutants, using a probe specific to the inserted Ll.ltrB intron. *Bsa*BI digests were performed on three clones of *CD2831* mutants and parent strain 630Δ*erm*. The digest for clone three was incomplete (left), leading to a faint 3.8 kb band on the Southern blot (right). L = HyperLadder I (Bioline), Lanes 1-3: Three mutant clones, W: wildtype 630Δ*erm*.


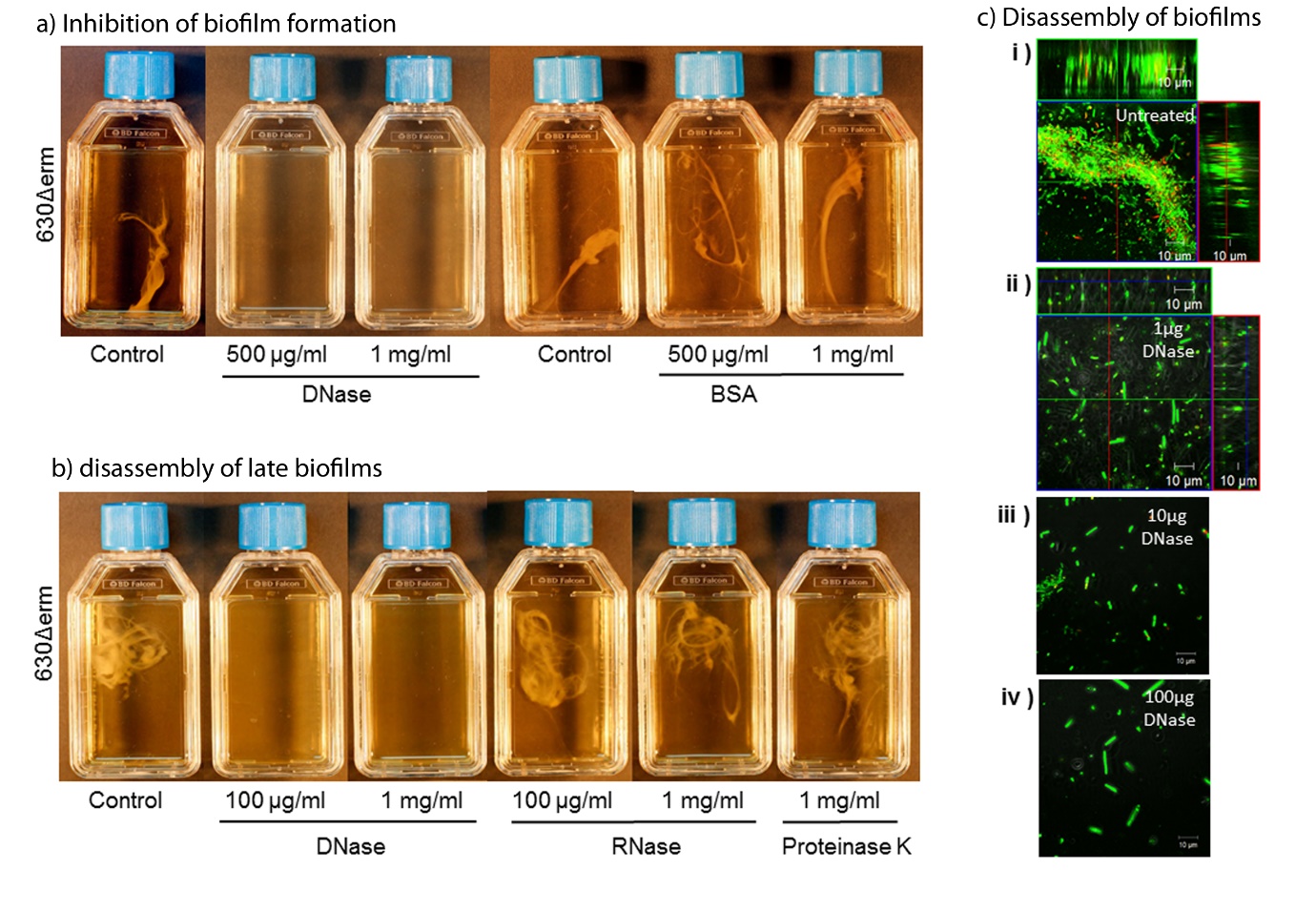


**Supplementary Figure 5: Biofilm disassembly and inhibition for *C. difficile* strain 630Δ*erm*.** a) Inhibition of biofilm formation: DNase (500 µg/mL and 1 mg/mL) or BSA (500 µg/mL and 1 mg/mL) was added to media before inoculation with *C. difficile* cells, flasks were then left for 72 hours to allow biofilm formation and maturation to occur. Late biofilms were detached from the bottom of the tissue culture flasks by gentle agitation, photographed using a Canon 450D, and complied in Photoshop Elements. b) Late biofilms were gently detached from the bottom of the tissue culture flasks by gentle agitation. DNase (1 mg/mL and 100 µg/mL), RNase (1 mg/mL and 100 µg/mL) or Proteinase K (1 mg/mL) were added for 15 minutes, and biofilms assessed by photography using a Canon 450D, and compiled in Photoshop Elements. c) Confocal microscopy visualisation of DNase treated biofilms (100, 10 and 1 µg/mL) compared to an untreated control. Biofilms were formed on Thermanox coverslips with strain 630Δ*erm* treated with or without DNase then stained with Film Tracer Live/Dead stain.


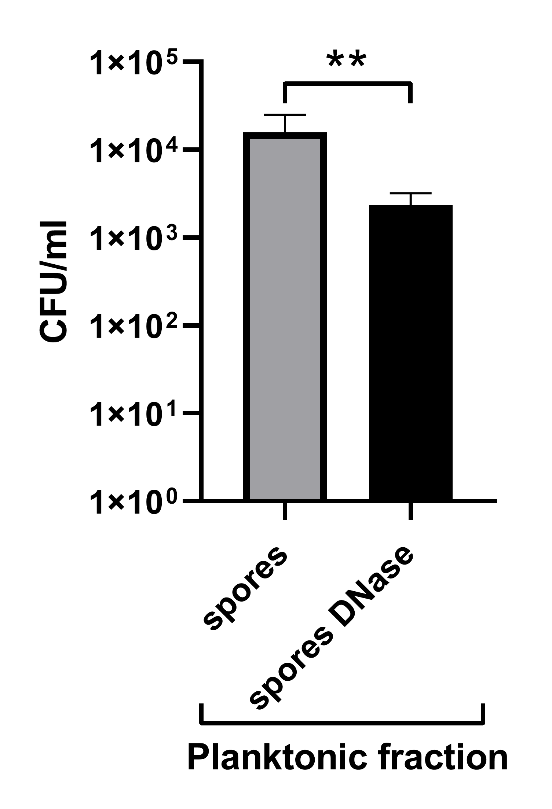


**Supplementary Figure 6: The effect of DNase on spore viability of planktonic biofilm fraction of *C. difficile* strain 630.** The effects of DNase on the viability of spores in the planktonic fraction of a biofilm compared to the untreated control. The planktonic fraction was left untreated or supplemented with recombinant DNase and spores were enumerated by heat inactivation of spore samples, thus killing the vegetative cells, enumerating spores alone. Experiments were undertaken with a minimum of 3 biological replicates. The data was analysed in Excel and GraphPad Prism 7.0, and error bars represent SD. Statistical analysis to determine the effect of DNase on spore viability was performed using a two tailed t-test, ** *p*< 0.01.

**
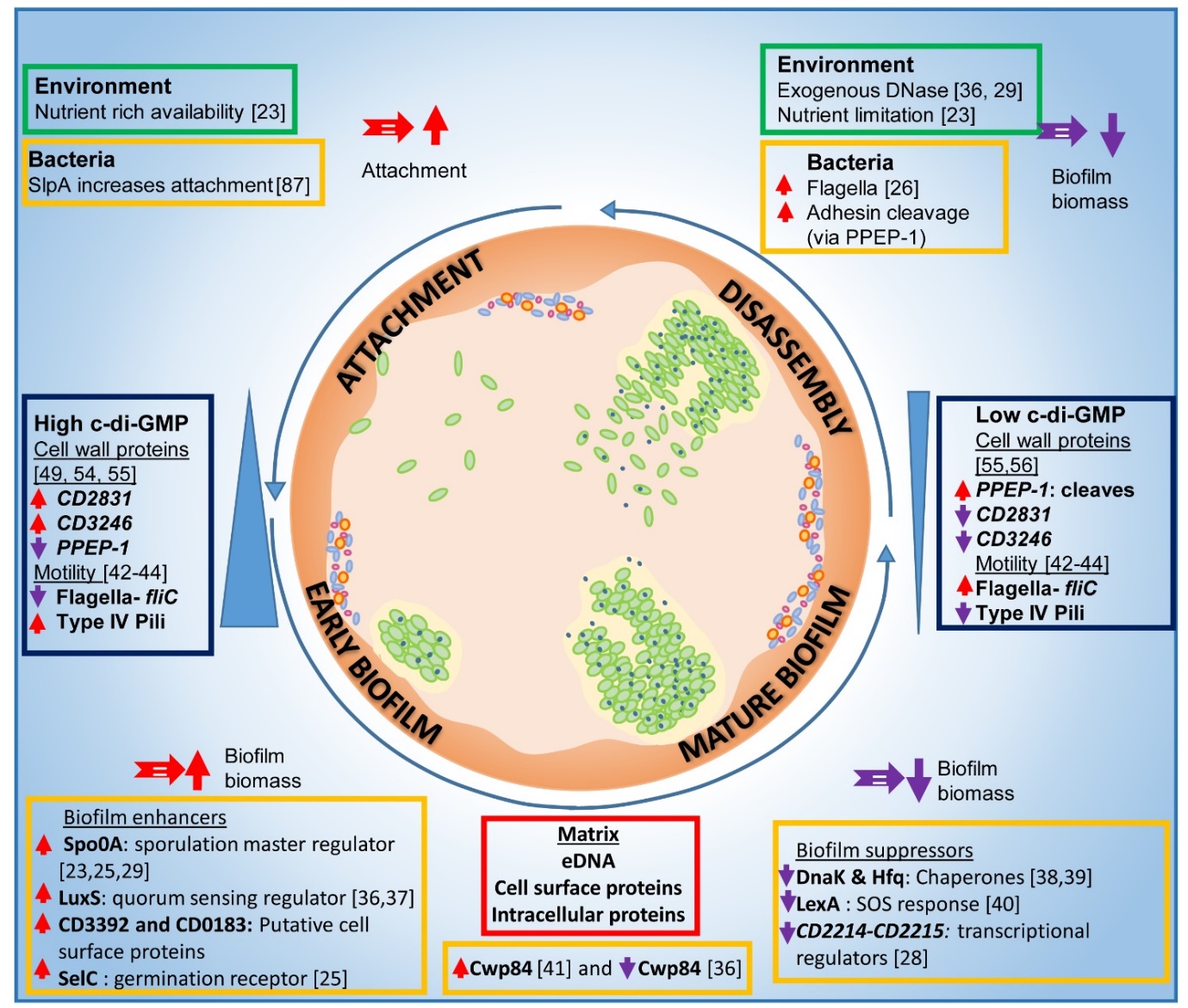
**

**Supplementary Figure 7: Collective summary outlining *C. difficile* biofilm formation.** The diagram shows the cycle of promoters of attachment, early biofilm formation, biofilm maturation and dispersal, from both this study and published data related to this work. Red arrows indicate an increase and purple arrows indicate a decrease in protein production and biofilm biomass. The blue triangles represent the level of c-di-GMP.


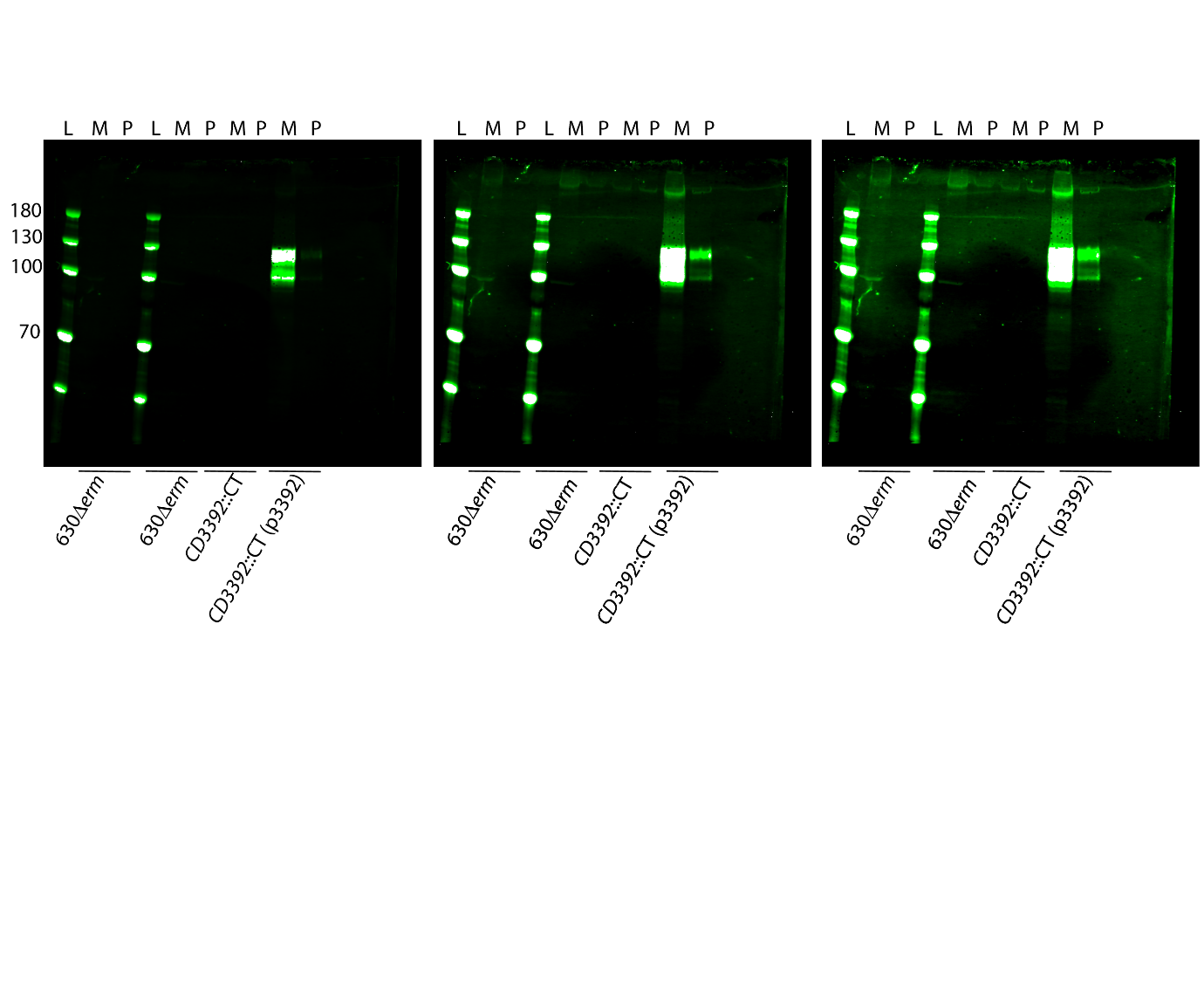


**Full length western blots from Fig. 6.** Western blot using anti CD3392 antibodies of the matrix (M) and planktonic fractions (P) of a late biofilm, from strains 630Δ*erm*, *CD3392*::CT and *CD3392* complement (*CD3392*::CT (p*CD3392*)). The biofilm matrix was detached from the bottom of a TC flask, disrupted by the addition of DNase (100 µg/mL), then loaded onto an SDS-PAGE gel alongside a protein ladder (L) for analysis by Western blot. We present three images scanned from low to high intensity from left to right. The ladder size markers are indicated on the left panel of the figure.
